# Preprocessing and Denoising Techniques for Electrocardiography and Magnetocardiography: A Review

**DOI:** 10.3390/bioengineering11111109

**Published:** 2024-11-02

**Authors:** Yifan Jia, Hongyu Pei, Jiaqi Liang, Yuheng Zhou, Yanfei Yang, Yangyang Cui, Min Xiang

**Affiliations:** 1 Key Laboratory of Ultra-Weak Magnetic Field Measurement Technology, Ministry of Education, School of Instrumentation and Optoelectronic Engineering, Beihang University, Beijing 100191, China; jyf@buaa.edu.cn (Y.J.); hongyupei@buaa.edu.cn (H.P.); ljq_vip77@buaa.edu.cn (J.L.); zhouyuheng@buaa.edu.cn (Y.Z.); yanfeiyang@buaa.edu.cn (Y.Y.); 2 Zhejiang Provincial Key Laboratory of Ultra-Weak Magnetic-Field Space and Applied Technology, Hangzhou Innovation Institute, Beihang University, Hangzhou 310051, China; 3 State Key Laboratory of Traditional Chinese Medicine Syndrome, National Institute of Extremely-Weak Magnetic Field Infrastructure, Hangzhou 310028, China; 4 Hefei National Laboratory, Hefei 230088, China

**Keywords:** Electrocardiography (ECG), Magnetocardiography (MCG), cardiovascular diseases (CVDs), signal preprocessing, denoising techniques

## Abstract

This review systematically analyzes the latest advancements in preprocessing techniques for Electrocardiography (ECG) and Magnetocardiography (MCG) signals over the past decade. ECG and MCG play crucial roles in cardiovascular disease (CVD) detection, but both are susceptible to noise interference. This paper categorizes and compares different ECG denoising methods based on noise types, such as baseline wander (BW), electromyographic noise (EMG), power line interference (PLI), and composite noise. It also examines the complexity of MCG signal denoising, highlighting the challenges posed by environmental and instrumental interference. This review is the first to systematically compare the characteristics of ECG and MCG signals, emphasizing their complementary nature. MCG holds significant potential for improving the precision of CVD clinical diagnosis. Additionally, it evaluates the limitations of current denoising methods in clinical applications and outlines future directions, including the potential of explainable neural networks, multi-task neural networks, and the combination of deep learning with traditional methods to enhance denoising performance and diagnostic accuracy. In summary, while traditional filtering techniques remain relevant, hybrid strategies combining machine learning offer substantial potential for advancing signal processing and clinical diagnostics. This review contributes to the field by providing a comprehensive framework for selecting and improving denoising techniques, better facilitating signal quality enhancement and the accuracy of CVD diagnostics.

## 1. Introduction

Cardiovascular diseases (CVDs) are the leading cause of death worldwide, with approximately 17.9 million people dying from them each year, according to the World Health Organization (WHO) report in 2023 [1]. Noncommunicable diseases claim the lives of approximately 17 million people under the age of 70. Of these deaths, 38% are due to CVDs [2]. Major CVDs include several conditions, such as congenital heart defects, coronary artery disease, cardiomyopathy, myocarditis, and myocardial infarction [3]. These diseases affect the structure and function of the heart, leading to potentially serious and life-threatening complications. Identifying individuals at the greatest risk of CVDs and providing them with effective treatment is crucial to reducing premature mortality. Various methods are used to detect CVDs, including Electrocardiography (ECG), echocardiogram, cardiac catheterization, cardiac computerized tomography scan, and cardiac magnetic resonance imaging [4].

**Figure 1:**
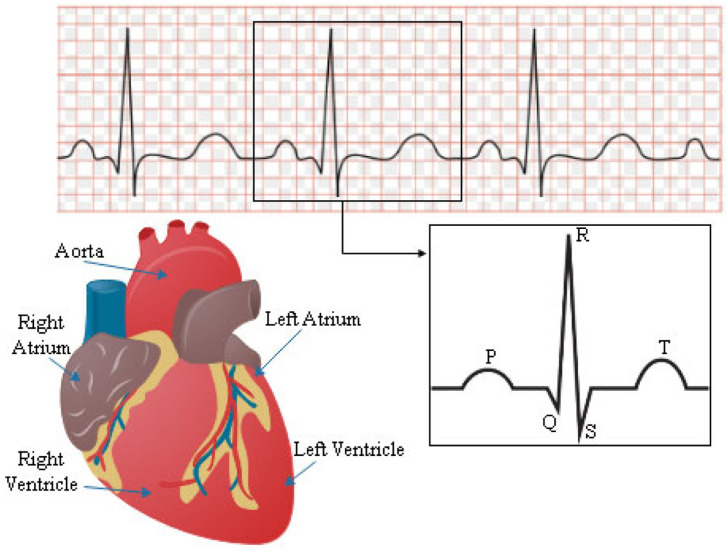
Tracing of the overall electrical activity of the heart [6].

The heart’s conduction system is responsible for producing and transmitting electrical signals that lead to the contraction of the heart muscle and the subsequent pumping of blood. These electrical impulses can be measured non-invasively by placing electrodes on certain areas of the body. The data obtained from these electrodes are then compiled into a composite graph, known as an ECG, which provides a detailed representation of the heart’s electrical activity [5]. The ECG waveform results from numerous action potentials propagated throughout the heart, as shown in Figure 1.

Over the past hundred years, the ECG emerges as an important diagnostic test for cardiovascular conditions and a crucial tool in modern clinical practice, following the development of the string galvanometer by Willem Einthoven [7]. Today, the ECG is the most frequently performed cardiovascular diagnostic test, and its widespread use is a testament to its significance in the field of cardiology [8,9]. However, due to the need for direct contact between the ECG electrodes and the human body during measurement, the signals are influenced by the body’s structure and conductive properties, leading to information loss. Additionally, ECG can only capture the electrical activity conducted to the body surface and cannot directly measure the heart’s contraction and relaxation activities, limiting its accuracy [10]. Like all electrical activities, the heart’s electrophysiological contractions also generate magnetic fields, which can be captured by a Magnetocardiography (MCG) [11,12].

Multiple studies show that MCG and ECG provide complementary information [13,14,15,16,17,18,19]. MCG is a highly precise multi-channel mapping technique that allows for the non-invasive, non-contact, and radiation-free recording of the electromagnetic activity of the heart. With resolution capabilities ranging from 10^−11^ Tesla to 10^−14^ Tesla, this method enables the accurate measurement and visualization of cardiac activity [11,20]. ECG and MCG signals are often subjected to various interferences and noise in practical applications. Therefore, denoising is crucial [21]. Effective denoising enhances signal quality and accuracy, aiding in the precise analysis of cardiac activity characteristics and anomalies. In clinical applications, this is essential for diagnosing and treating heart diseases, as even minor noise can obscure important signals. A robust denoising method provides three significant benefits for subsequent processing: (1) improved accuracy of feature extraction, (2) enhanced performance and reliability of computerized cardiovascular disease diagnostic systems, and (3) easier interpretation for clinicians [22].

ECG signal denoising methods receive extensive research and application. The existing literature comprehensively summarizes various denoising techniques, providing a systematic knowledge framework that allows researchers to understand the principles, advantages, disadvantages, and appropriate applications of current denoising technologies [4,23,24]. This enhances understanding and selection efficiency and promotes academic exchange and collaboration. However, to our knowledge, no literature systematically categorizes and compares ECG denoising methods based on noise types. Summarizing and comparing ECG denoising methods according to noise types specifically evaluates the effectiveness and applicability of different methods. Each noise type, such as baseline wander (BW), power line interference (PLI), electromyographic noise (EMG), or motion artifacts (MAs), has distinct characteristics and impacts. Different denoising methods vary significantly in their effectiveness against specific types of noise. By categorizing and comparing methods based on noise types, researchers and clinicians identify the performance strengths and limitations of each method in addressing specific noises, guiding them to select the most appropriate denoising techniques. Furthermore, this classification approach helps to identify deficiencies in existing denoising techniques, driving the development of more targeted new methods.

Denoising MCG signals presents greater challenges compared to ECG signals. First, the sources of noise in MCG measurements are complex, including environmental magnetic fields, electromagnetic interference from equipment, and the magnetic properties of the subject being measured. Second, MCG signals are more sensitive to noise, with any minor changes from the equipment, environment, or subject significantly impacting the signal, thereby increasing the difficulty of denoising. Additionally, the complexity and dynamic nature of MCG signals further complicate the denoising process [25,26,27]. The complexity and high cost of MCG equipment, along with the technical expertise required, limit related research and clinical applications. Currently, there is no standardized method for MCG denoising, and research findings are scattered, lacking a systematic review.

This review provides a comprehensive summary of recent advancements in denoising techniques for ECG and MCG signals, focusing on the strengths and limitations of each method. The review aims to guide researchers and clinicians in selecting appropriate techniques for specific noise types and signal preprocessing challenges. Data are sourced from IEEE Xplore, PubMed, and ScienceDirect databases, using the search terms “ECG/MCG & denoising/preprocessing”. Three independent researchers manually reviewed the studies to ensure the relevance of the included literature to the review’s objectives.

Studies are included if they propose new or significantly advanced denoising techniques for ECG or MCG signals, are published in peer-reviewed journals between 2013 and 2024, and report experimental results on denoising performance. This review focuses on papers that address ECG and MCG signal types, specifically targeting signal preprocessing and denoising methods. Included studies cover various noise processing techniques, such as BW, PLI, and EMG noise, employing methods like filtering, empirical mode decomposition (EMD), Kalman filtering, and convolutional neural networks (CNNs), among others. Only English-language publications are considered to ensure access to mainstream research. Excluded studies include those focusing on other signal types, lacking sufficient experimental results, or duplicating previously reviewed work. Abstracts, reviews, book chapters, and non-peer-reviewed articles are also excluded to ensure the quality of the selected studies. A total of 128 papers are reviewed, with 102 focusing on ECG denoising techniques, 18 on MCG denoising, and 8 on the relationship between ECG and MCG. Of these, 60 studies provide quantitative evaluations of denoising methods using metrics like signal-to-noise ratio improvement (SNR_imp_) and root-mean-square error (RMSE). The remaining 52 papers are selected for their contributions to new methods or explorations of real-time applications.

The structure of this paper is as follows: Section 2 describes the relationship between ECG and MCG signals; Section 3 introduces common noise types in ECG signals and their characteristics; Section 4 compares and presents ECG preprocessing techniques based on these noise types; Section 5 outlines the characteristics of noise in MCG signals; Section 6 systematically summarizes preprocessing techniques for MCG signals; Section 7 introduces evaluation parameters; Section 8 discusses challenges in preprocessing and denoising techniques; finally, Section 9 presents the conclusions.

## 2. Relationship Between MCG and ECG Signal

The magnetic field and electric current are closely correlated. Magnetic signals are less influenced by the inhomogeneous conductivity of bodily tissues than cardiac electrical signals since the permeability of the human body is constant. This makes them more dependable for the detection of biological occurrences [28].

ECG and MCG exhibit remarkable temporal resolution. However, the electrical conductivity in body tissues, particularly bones and fat layers, poses a challenge by acting as a low-pass spatial filter, thereby attenuating the electrical cardiac signals recorded in the ECG. This phenomenon results in a current flowing from a specific cardiac region, producing an ECG effect at almost all surface electrodes [29]. Therefore, the spatial resolution of ECG is notably inferior compared to MCG. MCG possesses an additional advantage over ECG as it can identify the magnetic field produced by intracellular and extracellular currents in cardiac tissue while ECG only registers the effects of currents flowing through body tissue [30,31]. Clinical trials have shown that MCG mapping is efficacious in characterizing and discriminating Brugada syndrome and complete right bundle branch block and in identifying spatial current dispersion patterns [32]. MCG mapping has been found to offer superior statistical sensitivity compared to ECG, thereby providing an opportunity for more accurate diagnosis of cardiac arrhythmia and coronary artery diseases [11,33]. Through the inverse problem, the measured multi-channel ECG/MCG signal can be combined with the signal transmission model to characterize the activity of the heart, that is, non-invasive cardiogenic imaging [34,35].

MCG and ECG may provide complementing information, according to several research studies [14,15,16,17,18,19,36]. Several interpretations have suggested that the sensitivity of MCG and ECG differs depending on the source configuration: MCG has been considered as more sensitive to “tangential” [14,17,18] and “vortex” [16,36,37] currents, whereas ECG has been considered as more sensitive to “radial” [14,17] currents. However, it is not clearly defined whether the term “current” refers to cardiac activation (primary current) or the total current close to the body surface and sensors. Also, the terms “radial” and “tangential” are not clearly defined. Sometimes they refer to the direction of the current relative to the heart, and sometimes they refer to the direction of the current relative to the ECG/MCG sensor or body surface [13].

**Figure 2:**
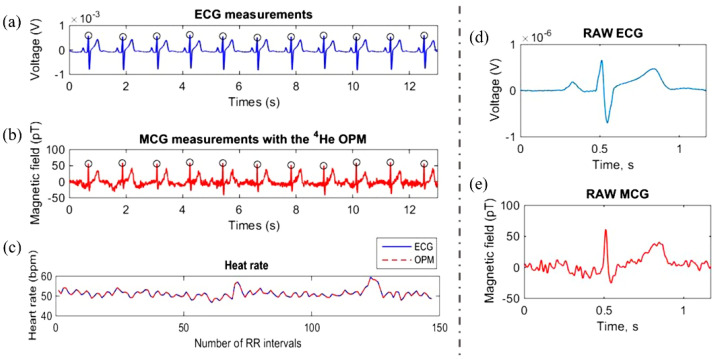
Real-time cardiac signal of a healthy subject detected simultaneously with (**a**) ECG electrodes and (**b**) MCG optically pumped magnetometer (OPM); (**c**) heart rate determined from simultaneous ECG and OPM recordings; ECG and MCG signals of a healthy volunteer. Real-time filtered un-averaged (**d**) ECG signal and (**e**) MCG signal with one heart beat cycle. Figure is modified from [29].

S. Morales et al. simultaneously conduct ECG and MCG examinations on healthy individuals [29]. The recorded ECG and MCG data are shown in Figure 2a,b. The QRS complex and T wave are clearly visible in both datasets. Heart rates (determined based on RR intervals in ECG and MCG data) completely overlap, as depicted in Figure 2c. This confirms that there is no statistical difference between the heart rates determined by ECG and MCG. Additionally, there are no statistical differences observed in the QRS duration, QT interval, and corrected QT interval between ECG and MCG. The signal of a single cycle of filtered ECG/MCG is shown in Figure 2d,e. Due to the weak signal and sensor sensitivity, MCG is more susceptible to external magnetic field interference and exhibits greater noise compared to the ECG signal. The types and characteristics of noise in MCG/ECG signals, along with commonly used denoising methods, are analyzed in detail in the following sections.

## 3. Noises in ECG Signal

The morphological and interval features in recorded ECG signals serve as crucial indicators for evaluating cardiac health outcomes. Nevertheless, ECG signals are prone to diverse predominant noises, including BW, EMG/MA, channel noise (additive white Gaussian noise, AWGN), PLI, and miscellaneous noises like composite noise, random noise, electrode motion artifacts (EMs), and instrumentation noise. These diverse interferences pose a formidable challenge in discerning disease-specific morphological anomalies within ECG signals.

**Figure 3:**
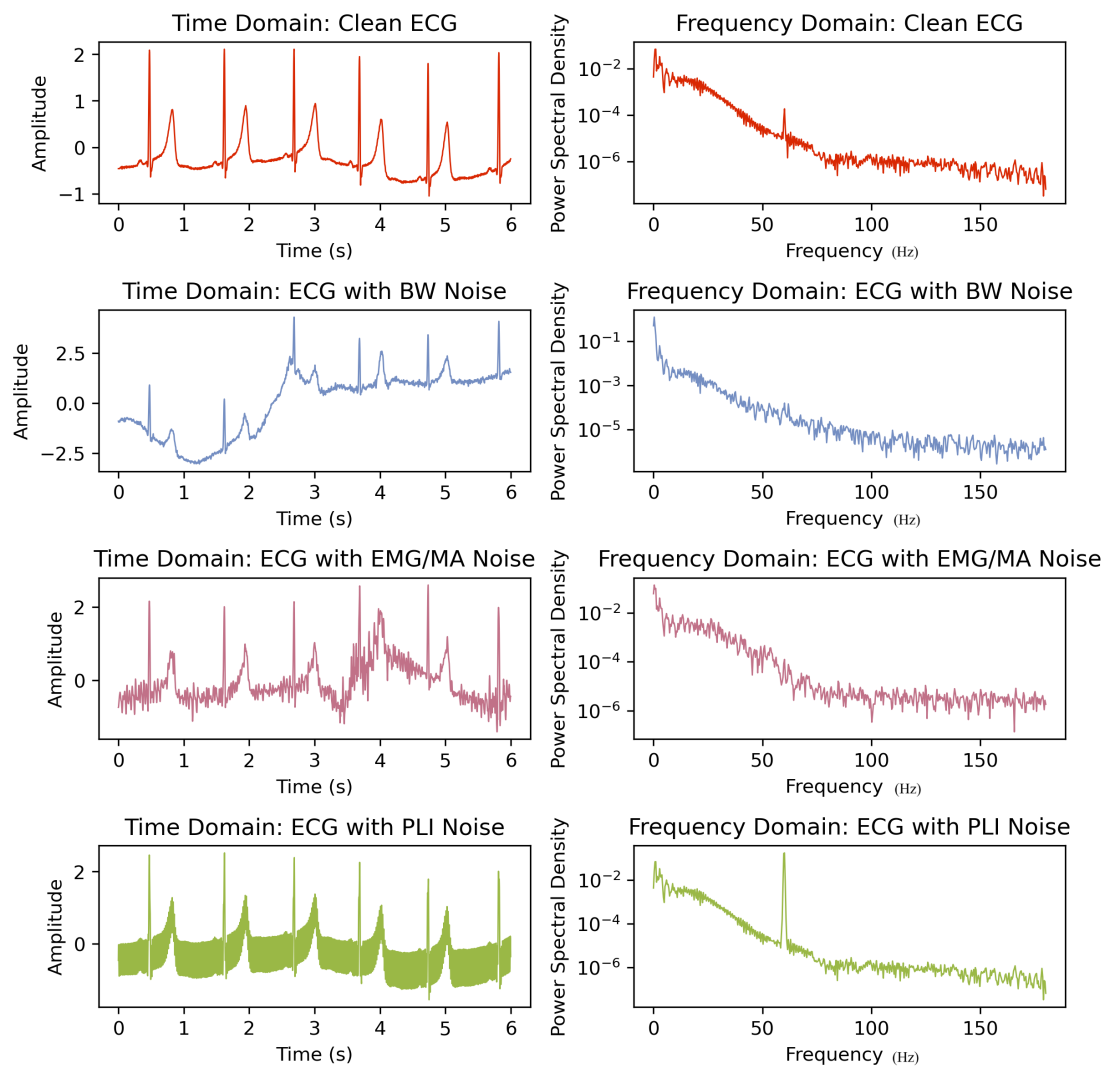
Time-domain and frequency-domain representations of ECG signals with BW, EMG/MA, and PLI noise components.

BW, the addition of a sinusoidal component at the frequency of respiration, is a common occurrence in ECG signals [38]. BW is a low-frequency artifact and can be caused by breathing, subject movement, or electrically charged electrodes. Its amplitude is typically around 5% and occurs at frequencies ranging from 0.15 Hz to 0.3 Hz. This is often caused by the patient’s respiration, and can also be simulated by adjusting frequency and amplitude values in Equation (1). The spectrum of the ECG with BW noise is depicted in Figure 3. In the spectrum, variations below 1 Hz are attributed to BW effects. Abrupt baseline drift, which arises from patient movement and changes in skin–electrode impedance during ECG measurement, can be simulated by adding a DC bias to the ECG signal for a chosen period.

EMG/MA noise is a type of interference in ECG signals that typically occurs in the frequency range of 5 to 500 Hz. This type of noise results from muscular contraction and relaxation. The EMG signal is regarded as one of the most prominent artifacts in wearable ECG recordings. The identification of EMG noise presents a greater challenge compared to PLI and BW noises, given its non-stationary nature and substantial overlaps across entire frequency bands with ECG data, as shown in Figure 3. This noise generates disturbances with an amplitude equivalent to 10% of a standard peak-to-peak ECG. The disruptions caused by EMG noise result in alterations to the structural integrity of the recorded signal, thereby complicating the processes of diagnosis and interpretation [39]. In the simulation experiment, colored noise is generated to simulate EMG by subjecting white noise to bandpass filtering. Specifically, the cutoff frequency of the filter is set within the range from 5 Hz to the minimum of the sensor cutoff frequency *f_c_* or 500 Hz.

Electromagnetic interference caused by power lines can result in PLI in ECG signals [38]. PLI is produced by the interaction between the data cables that carry the ECG signals from the patient to the display devices and the 50/60 Hz power line noise. The presence of this noise cannot be totally prevented, despite the device having a very high common mode rejection ratio [40]. This type of interference is highly consequential in ECG signals, as it can impair both the quality and finer characteristics of the signals, which are vital for accurate clinical diagnosis. PLI noise $N_{PLI}\left(t\right)$ can be simulated with the following sine function:

(1) $$\begin{array}{c} N_{PLI}\left(t\right)=A_{PLI}\times sin\left(2\pi f_{PLI}\cdot t\right) \end{array}$$

where $A_{PLI}$ denotes the amplitude of the power line. $f_{PLI}$ denotes the frequency of the power line; usually, the value is 50 Hz or 60 Hz.

BW and PLI are two types of signal disruptions that affect ECG signals differently. BW affects low-frequency signals while PLI affects high-frequency signals. Both types of disruptions can change important characteristics of ECG signals. BW disturbs the ST segment period, which is critical for diagnosing CVDs [41]. PLI can also disrupt the P and T waves of an ECG signal, making interpretation difficult [42]. To address these issues, it is necessary to remove these noises without modifying the ECG signal’s properties. AWGN is a type of channel noise that can affect ECG signals when they are transmitted over channels with poor conditions, such as high levels of AWGN [43]. Combining two or more of the aforementioned noises is referred to as composite noise [44]. Gaussian white noise possesses randomness and statistical properties, making it capable of simulating many noise sources found in the real world. In most studies on ECG denoising, Gaussian white noise is commonly employed to simulate composite signals.

## 4. Pre-Processing in ECG Signal

Although there are many machine learning methods to choose from, choosing the appropriate methods and parameters remains a challenging issue. Different types of noise may correspond to different optimal numbers of neurons, which means that we need to adjust the structure and hyperparameters of the neural network based on specific noise types and datasets. Some researchers compare the effects of different methods on different types of noise through experiments and attempt to find the optimal number of neurons [45].

### 4.1. BW Removal in ECG Signal

The challenge of eliminating BW primarily stems from the significant overlap between the BW spectrum and the ST segment, which can lead to various distortions in ECG signals. Large BW can cause issues such as peak clipping by recording instrument amplifiers, alterations in the shape and duration of the ST segment, and the loss of low-amplitude peaks like P waves and T waves [46]. The most widely used approach to removing BW is the application of a high-pass filter [47]. However, this method often leads to computational delays and ringing artifacts due to the filter’s long response time. While high-order IIR filters have been employed to mitigate these effects [48,49], they introduce problems like phase distortion and a long filtering process. Zero-phase bi-directional filters, which address these two issues, have been proposed as an alternative [50]. However, the precise tuning of filter bandwidth remains challenging, and high-pass filters can result in the loss of low-frequency components, further distorting the ST segment.

**Figure 4:**
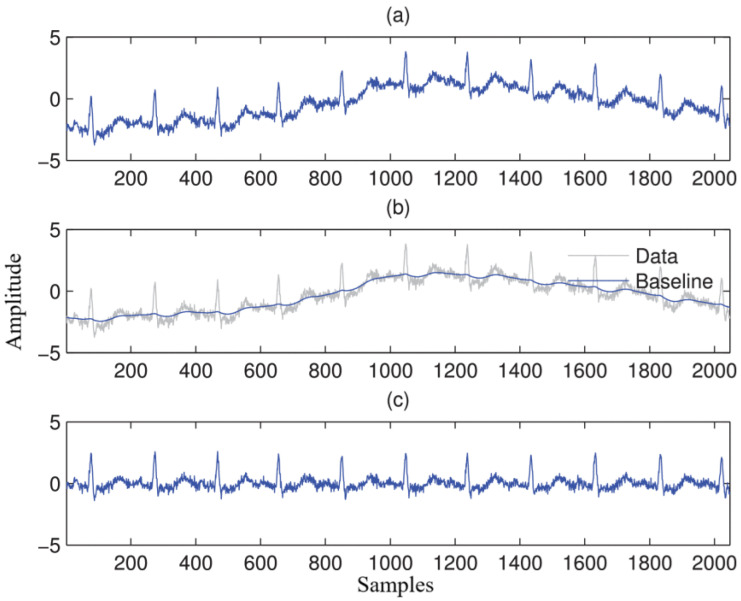
Baseline wander correction. (**a**) The ECG signal added BW signal and noise. (**b**) Baseline estimation. (**c**) The ECG signal after BW correction. Figure is modified from [51].

Some researchers have explored hybrid methods that combine sparse signal modeling with digital filtering to estimate and remove the baseline [51,52], as shown in Figure 4. Although these methods improve flexibility in filter design, they still face challenges in maintaining signal integrity, especially regarding parameter selection and the potential introduction of distortions. Another commonly used approach is the cubic spline method, which estimates BW under the assumption that the PR segment is correctly identified and accurately detected for normal beats [53]. However, noise from sources such as electrode motion and muscle artifacts often obscures the PR segment, leading to limited effectiveness [54].

Wavelet decomposition has become a widely studied method for removing BW, as it allows for multi-scale analysis of the ECG signal [55,56,57,58]. The process involves decomposing the signal into different frequency scales and using the inverse wavelet transform to reconstruct a denoised signal. Although wavelet-based methods are effective, automating the identification of BW components remains difficult because baseline noise can be distributed across multiple wavelet functions. In response to this, some researchers have proposed Fourier decomposition (FD) to simultaneously remove BW and PLI [40]. This method decomposes the signal in the frequency domain, providing clean ECG data, though it is limited by its reliance on frequency-domain assumptions.

Projection-operator-based techniques have also been proposed to address BW removal by separating the noise from the ECG signal [46,59]. These methods perform well in low-noise environments but struggle when multiple types of interference are present. Another increasingly popular approach for BW removal is EMD, which decomposes the signal into intrinsic mode functions (IMFs) based on its time-scale characteristics [60,61,62]. EMD has excellent adaptability but is prone to mode mixing, which can compromise the quality of the denoised signal. To counteract this, enhanced methods like EMD combined with polynomial fitting [63] and low-pass filtering [61] have been proposed. Other studies combine EMD with morphological filtering [64], while some utilize the Hilbert transform for improved decomposition accuracy [65]. Additionally, complete ensemble EMD with adaptive noise (CEEMDAN) has been introduced to improve BW correction, incorporating wavelet thresholding for enhanced performance [66].

Other widely used techniques include adaptive filtering and moving average filters. Adaptive filtering, which requires a reference signal, has been successfully employed for BW removal [67,68]. However, obtaining an accurate reference signal can be difficult, limiting its practicality. Moving average filters are computationally efficient and easy to implement [69], but they can distort the signal when there are abrupt changes in amplitude, especially near R-peaks. Despite these drawbacks, EMD remains a popular choice due to its superior time-scale resolution.

With the recent advancements in deep learning, neural-network-based methods are increasingly applied to ECG denoising tasks. Techniques such as autoencoders [70], generative adversarial networks (GANs), and variational autoencoders are proving effective in removing low-frequency noise like BW. Research has shown that increasing the number of neurons in these networks can enhance their ability to suppress BW noise [45]. However, while adding more neurons increases the network’s capacity to model and remove noise, it also introduces complexity, which can reduce its effectiveness in handling composite noise.

Table 1 and Table 2 present a comparison of BW denoising techniques for ECG signals. Among the standout methods, the dual-tree wavelet transform [71] and biorthogonal wavelet transform [72] demonstrate strong performance, with dual-tree wavelet transform achieving an SNR improvement of 15.25 dB and low MSE, and biorthogonal wavelet transform showing an impressive SNR of 28.38 dB. These methods excel in noise reduction with minimal signal distortion. Deep learning approaches also perform well, such as the DNN-based DAE with wavelet transform [70], which balances a high SNR of 23.89 dB with low RMSE, and the U-Net model [73], which offers robust noise suppression, with an SNR of 19.51 dB and RMSE of 0.0132. The bidirectional GRU method [74] offers another effective solution, with an SNR improvement of 20.6 dB and PRD of 5.5%, showing a good balance between noise reduction and signal fidelity. In summary, while traditional filtering techniques are computationally efficient, they can introduce signal distortion. Advanced methods like wavelet decomposition, EMD, and deep learning provide greater precision but pose challenges such as complexity and automation. Hybrid methods that combine multiple approaches are likely to emerge as the most effective solutions for BW denoising in ECG signals.

### 4.2. EMG/MA Removal in ECG Signal

The primary challenge in eliminating EMG noise from ECG signals lies in its dynamic nature [84]. Various methods address EMG noise, ranging from traditional filtering techniques to advanced statistical and morphological approaches. One widely used method is discrete wavelet transform (DWT), which employs multi-resolution analysis for real-time noise reduction [58]. Studies suggest that segmented processing is more effective than applying uniform thresholds across the entire signal, especially when using hard and soft thresholding functions, which enhance denoising efficiency. However, wavelet-based techniques often struggle to preserve sharp features, such as QRS complexes, and may introduce distortions when there is spectral overlap between the signal and noise [27,85,86,87,88]. To improve performance, Xiong et al. propose a cosine transform least squares (LMS) adaptive cancellation algorithm (TDC-LMS) that demonstrates superior performance over the classical LMS algorithm in eliminating high-amplitude motion artifacts [89]. However, this adaptive approach requires an additional noise-correlated reference signal, complicating hardware design and limiting its applicability for real-time analysis.

**Figure 5:**
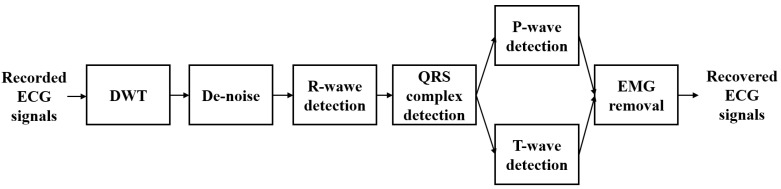
The flowchart of the denoising method for ECG delineation system based on DWT.

Morphology-based methods, which use the structural characteristics of ECG signals as prior information, also exist. Lin et al. utilize ECG morphological features to remove EMG noise, as illustrated in Figure 5, showing significant effectiveness in low-noise environments [90]. Similarly, Li et al. introduce a non-local means (NLM) filtering approach, known as periodic NLM (pNLM) filtering, which reduces noise even when EMG and ECG signals overlap in frequency bands [91]. However, this method’s performance decreases in cases of periodic deviations, such as premature contractions, atrial fibrillation, or motion artifacts [92].

Kalman filtering techniques, particularly the extended Kalman filter (EKF), are also employed for noise removal, utilizing the morphological characteristics of ECG signals. Hesar and Mohebbi propose the marginalized particle EKF (MP-EKF), a model-based Bayesian filtering framework suitable for non-Gaussian and non-stationary signals [93]. Despite its effectiveness, MP-EKF is less suitable for signals with sudden morphological changes, such as those during arrhythmias. To address this, Hesar and Mohebbi introduce a fuzzy adaptive particle weighting strategy within the Bayesian framework to maintain performance despite deviations from predefined signal models [94]. Additionally, they present an adaptive Bayesian method that does not require a predefined model, significantly reducing preprocessing efforts by only needing R-peak positions [95]. Akhbari et al. propose another EKF-based method for both denoising and extracting fiducial points of ECG signals [96]. This method requires manual initialization of parameters related to the amplitude, width, and phase of each component of the cardiac cycle. However, its performance significantly decreases when the ECG signal deviates from the model, such as during arrhythmias.

Mourad et al. present a successive local filtering algorithm (SLFA) for denoising ECG signals contaminated by EMG [97]. SLFA segments the recorded ECG data, applying ideal filters to remove wideband noise while preserving signal integrity, adapting the filter bandwidth to match the dominant component in each segment. Hybrid methods, such as the approach by Rakshit et al., integrate EMD with adaptive stationary morphological filtering (ASMF). This method first applies wavelet-based soft thresholding to eliminate high-frequency noise, followed by ASMF to enhance the signal, balancing denoising with signal preservation [98]. Statistical techniques such as independent component analysis (ICA) and principal component analysis (PCA) have proven effective for separating EMG noise from ECG signals [99,100]. While effective in multi-channel ECG settings, these methods require visual inspection of independent components, limiting their practicality for long-term applications. To improve this, Malghan et al. introduce a combination of variational mode decomposition (VMD) and the grasshopper optimization algorithm (GOA), which identifies relevant IMFs and enhances denoising performance [39]. Heydari Beni et al. conduct a comparative study of five improved EMD/DWT methods, showing that denoising performance varies with changes in environmental noise levels. This emphasizes the need to assess potential artifact levels before selecting a denoising method in practical applications [101].

ECG signal denoising for removing EMG/MA is summarized in Table 3. In the table, several denoising methods stand out for their strong performance. The U-Net model [73] achieved an SNR improvement of 16.76 dB and a low RMSE of 0.0182, indicating effective noise suppression with minimal signal distortion. Similarly, the CNN combined with a transformer encoder [82] reported a high SNR improvement of 28.11 dB, although the RMSE of 0.021 suggests that some signal distortion may occur. Both methods demonstrate the strength of deep learning in handling complex noise, though further refinement could help in preserving sharp ECG features. The bidirectional gated recurrent units (GRUs) approach [74], with an SNR improvement of 19.2 dB and a low PRD of 6.4%, offers a good balance between denoising and signal preservation, making it particularly well suited for practical applications. Though the SLFA [97] showed a lower SNR improvement (>8 dB), it remains effective in reducing noise, though detailed metrics like RMSE or PRD are needed for a fuller comparison. Overall, methods like GRU and U-Net offer promising solutions for denoising, particularly in scenarios requiring both real-time performance and high signal fidelity.

### 4.3. PLI Removal in ECG Signal

The main challenge in eliminating PLI lies in its frequency drift. The frequency is not fixed at 50 Hz or 60 Hz but fluctuates by approximately ±1 Hz. Various techniques have been developed to address this issue in ECG signals, each with distinct advantages and drawbacks.

**Figure 6:**
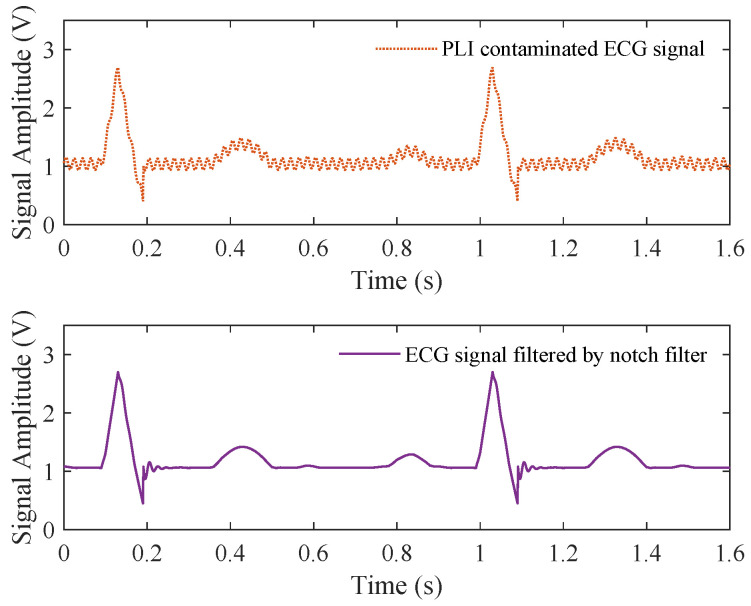
The ringing artifact after QRS complex filtered by notch filter.

A conventional approach involves applying notch filters, which are typically realized as either finite impulse response (FIR) or infinite impulse response (IIR) filters. IIR notch filters are often preferred due to their lower filter order compared to equivalent FIR filters. The ideal notch filter has a flat frequency response, except at 50 or 60 Hz, where it ideally yields a zero-frequency response. However, this is difficult to achieve in practice. Notch filters suffer from two key drawbacks. First, the frequency response may not be perfectly flat near the attenuated frequency, leading to a ringing artifact, particularly visible after the QRS complex, as shown in Figure 6 [105,106]. Zero-phase bi-directional filters have been developed to mitigate this issue [50]. Second, the fixed central frequency of notch filters makes them ineffective when PLI fluctuates, as shown in Figure 3, rendering them unsuitable for cases where frequency drift occurs [105]. Expanding the notch filter’s range to cover a wider frequency band can attenuate desired signals, making it impractical in many cases [107]. Moreover, notch filtering can distort the underlying cardiac signal, leading to erroneous diagnostic results [108].

To overcome the limitations of fixed-frequency filters, researchers have explored techniques based on signal decomposition. FD is one such method that has been widely adopted. FD divides the ECG signal into distinct frequency bands, and the frequency band containing PLI is removed entirely [109,110,111,112]. While computationally efficient, this approach leads to significant signal loss because it eliminates an entire band, affecting the quality of the denoised ECG signal. Similarly, EMD and its variants decompose the ECG signal into IMFs, with PLI being isolated in specific IMFs [113,114,115]. The removal of these IMFs may also erase essential cardiac features. Wavelet transform (WT)-based methods, including stationary wavelet transform (SWT), have also been applied to PLI removal [6,116,117]. However, WT struggles with edge detection, and choosing an appropriate basis function is complex [4]. Eigenvalue decomposition (EVD) methods estimate the eigenvectors corresponding to PLI interference and remove them from the signal [77]. However, this too can result in the loss of critical cardiac information.

Adaptive filtering has emerged as an effective alternative for dynamic PLI removal. Adaptive noise cancellation (ANC) adjusts its response based on input parameters such as amplitude, phase, and frequency [118,119]. ANC uses a reference signal to dynamically track PLI, typically relying on filters such as the LMS and recursive least square [120,121]. However, the need for a reference signal increases device complexity, making it challenging for portable systems. In response, adaptive sinusoidal interference canceller techniques are introduced, assuming that the PLI frequency is known while treating amplitude and phase as adaptive parameters [122]. Other methods, focus on isolating linear segments of the ECG for PLI estimation, though this necessitates prior detection of QRS complexes, adding complexity to the system [123].

State-space adaptive filtering methods have also been employed for PLI removal without requiring a reference signal. This approach is effective in non-stationary environments, where PLI frequency and amplitude vary. For example, Tahir et al. introduce an EKF that tracks and removes PLI in real time based on frequency fluctuations [124,125]. Despite its advantages, the EKF method is sensitive to the availability of accurate noise covariance information, and its performance degrades when this information is inaccurate. An alternative state space filter, proposed by Razzaq et al., eliminates both PLI and its harmonics without a reference signal, dynamically adjusting its tracking frequency to filter the first, third, and fifth harmonics of PLI [126]. However, this introduces significant computational overhead due to the need to filter multiple harmonic frequencies. Rehman et al. introduce a parallel-distributed version of state-space least mean squares with adaptive memory that improves performance while reducing computational load compared to sequential filtering methods [108]. Other innovative approaches include Qaisar’s signal-driven linear phase filtering technique, which efficiently compresses the signal while removing PLI and baseline wander with reduced computational costs [127]. Additionally, Chen et al. introduce a method combining notch filtering with modulation-based detection and frequency estimation, adjusting the filter’s notch frequency based on detected PLI characteristics [128].

In recent work, a variable notch filter designed via VMD is introduced by Mir and Singh, offering an effective method for reducing PLI in ECG signals [129]. Similarly, complex demodulation algorithms such as VFCDM have shown superior performance in filtering PLI compared to traditional methods like EMD and wavelet transforms [59]. Malghan and Hota propose an advanced variational mode extraction (VME) technique that integrates a heap-based optimization (HBO) algorithm for precise PLI removal. The HBO algorithm optimizes the extraction of the mode containing PLI, achieving improved signal quality in the denoised ECG [130]. Jain et al. introduce iterative multi-resolution techniques to enhance denoising performance by adjusting wavelet coefficients [131]. Hybrid techniques, such as the combination of adaptive and EMD-based methods, further enhance PLI removal [132]. Additionally, harmonic wavelet filtering techniques have also been explored to remove harmonics of PLI and retain the signal [133].

Table 4 summarizes the denoising methods for PLI denoising in ECG signals. The biorthogonal wavelet transform with adaptive slope prediction-based thresholding [72] achieves a high SNR of 30.01 dB with a very low MSE of 0.0008, showing excellent noise suppression with minimal signal distortion. Similarly, singular spectrum analysis combined with digital filtering [22] performs well, achieving an MSE of 0.000007, making it one of the most precise approaches. Other techniques like SWT [6] also perform well, with an SNR improvement of 49.35 dB, though some minor signal distortion may occur. In summary, while traditional notch filters are simple, they struggle with frequency drift and ringing artifacts, necessitating more advanced methods. Techniques such as decomposition, adaptive filtering, and state-space methods offer better noise rejection but come with higher complexity and computational demands. The trend is moving toward more adaptive and efficient PLI removal solutions, particularly for real-time and portable ECG monitoring systems.

### 4.4. Composite Noise Removal in ECG

Different types of noise exhibit distinct dynamic characteristics. Effectively removing these noises necessitates consideration of their interactions and complex composite effects. Researchers have developed various methods to address composite noise. These methods can be broadly categorized into three groups based on their underlying techniques and processing strategies: model-based methods, learning-based methods, and hybrid methods. Table 5 and Table 6 summarize the removal of the composite noise in the ECG signal. Model-based denoising methods utilize prior knowledge or models to describe the characteristics of ECG signals and then utilize these features for denoising. Common model-based denoising methods include filter design [103], NLM filtering [91], wavelet transform [104], EMD decomposition [135], and methods based on sparse representation [136].

**Figure 7:**
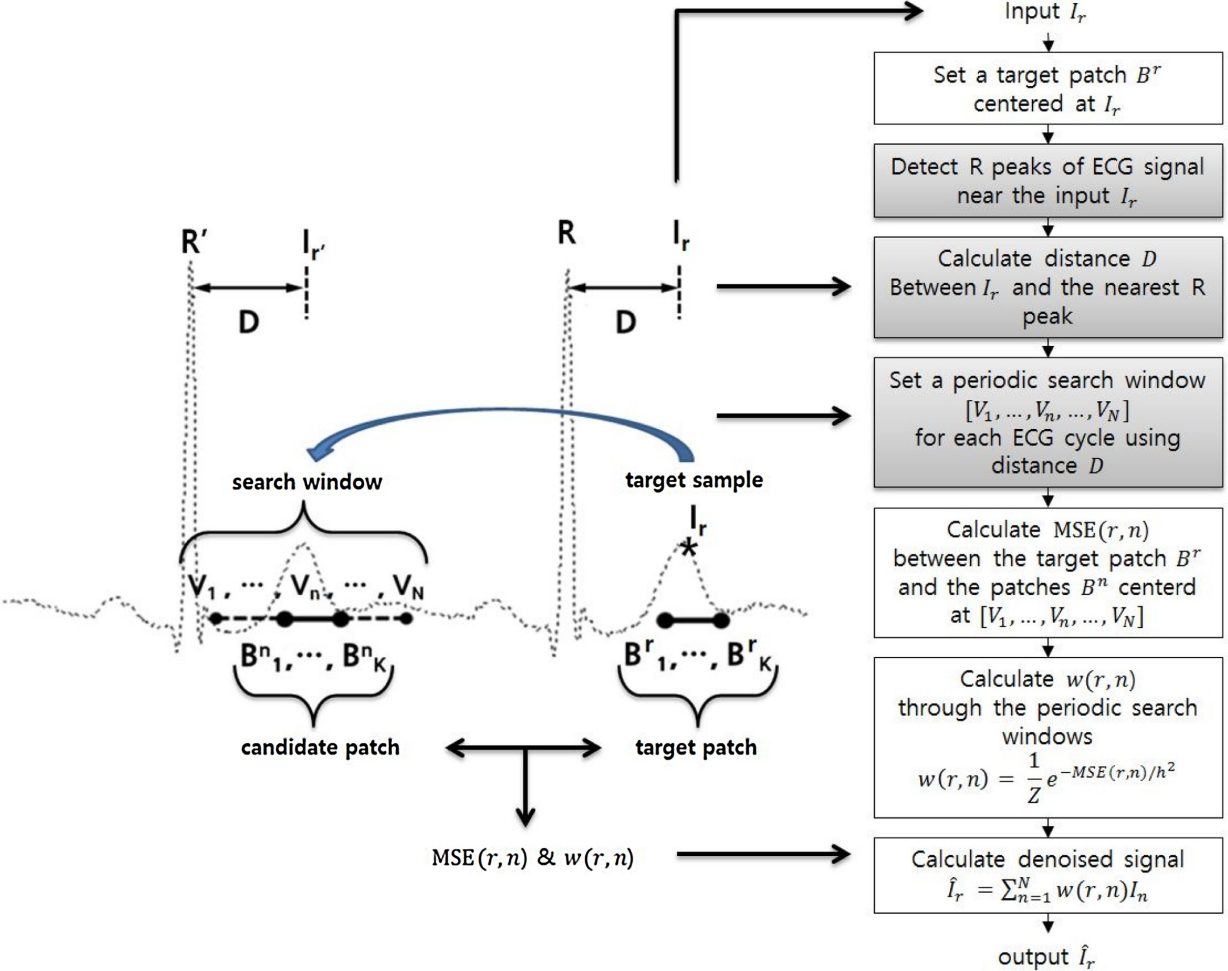
The illustration of the pNLM algorithm parameters and workflow [92].

The periodic structure of ECG signals makes them well suited for NLM denoising, with Lee et al. developing a pNLM filter that exploits this periodicity to enhance performance, though it requires accurate R-wave detection and incurs high computational costs [92], as shown in Figure 7. To address these challenges, Qian et al. introduce a local means method to reduce computational complexity [137]. Liu et al. propose a method that improves denoising by using collaborative filtering of similar segments, integrating both local and non-local information, though this approach faces high time costs and potential issues with segmentation accuracy [138].

Wavelet transform provides an alternative to traditional Fourier and short-time Fourier transforms by offering time–frequency components at any moment. It provides better time resolution at high frequencies and better frequency resolution at low frequencies. Commonly used wavelet techniques include DWT and SWT. Key factors for effective denoising are the selection of appropriate wavelet bases and thresholding methods. Azzouz et al. combine particle swarm optimization with wavelet transform to optimize ECG signal denoising parameters, including wavelet basis functions, thresholding functions, decomposition levels, and threshold selection rules [117]. Similarly, Kumar et al. compare the denoising performance of different wavelet bases and identify biorthogonal 3.1 as the most suitable for ECG signals [72,139]. Further, Upadhyay et al. explore the use of wavelet bases designed from the Schrödinger equation, which yield excellent results for ECG denoising [38]. The optimized ANC filter dynamically adjusts its coefficients based on the input signal to eliminate unwanted noise and artifact components effectively [140]. While many studies focus on the development of threshold selection methods for wavelet-based denoising [71,104], DWT presents certain limitations, including boundary effects, lack of translation invariance, and aliasing. SWT, on the other hand, better addresses boundary effects, and the dual-tree complex wavelet transform enhances anti-aliasing and translation invariance, making it particularly useful for biomedical signals [141]. To mitigate the loss of key information caused by thresholding low-frequency components in the DWT domain, some researchers combine DWT with NLM estimation for improved denoising performance [142,143]. Additionally, Madan et al. propose the stationary wavelet total variation method, which integrates total variation with bivariate shrinkage rules in the SWT domain. This approach maintains the original signal amplitude, making it suitable for clinical applications [102].

Wavelet-based techniques, while effective, can suffer from limitations such as boundary effects, lack of translation invariance, and aliasing. To address these issues, Kalman filters (KFs) have emerged as strong alternatives for ECG signal denoising. KF-based techniques enhance interpretability and reliability by leveraging state-space models that dynamically estimate and correct noisy signals based on prior information [144,145,146,147].

EMD presents a flexible alternative by adaptively decomposing signals into IMFs without requiring prior information. However, EMD methods often struggle with mode mixing, which reduces denoising effectiveness. Nguyen et al. propose an adaptive approach to mitigate this issue [148]. Jain et al. further enhance EMD by combining it with RL fractional integral filtering and SG filtering to remove various noise types [76], while Kumar et al. integrate EMD with NLM filtering to better preserve ECG signal edges [149]. Rakshit et al. propose a hybrid approach using EMD and adaptive switching median filtering (ASMF) for more robust noise reduction [98], and Wang et al. enhance EMD with ICEEMD to effectively denoise chaotic signals [150]. These methods offer flexible and adaptive approaches to ECG denoising, addressing some of the key shortcomings of wavelet-based techniques and expanding the toolkit for handling various noise types in biomedical signals.

Several new techniques for ECG denoising have been developed in recent years. Hossain et al. propose the VFCDM algorithm, which decomposes ECG signals into sub-bands to remove noise while dynamically reconstructing the signal based on noise severity [59]. Sharma et al. introduce an eigenvalue decomposition method that efficiently handles baseline drift and power line interference [77]. Tan et al. present a Blaschke unwinding adaptive FD approach that balances signal compression with effective denoising [151]. Bajaj et al. offer the fractional Stockwell transform, which excels at analyzing non-stationary signals but faces computational complexity issues [79]. These methods enhance specific aspects of ECG denoising, though they can introduce challenges such as complexity or limited applicability. In contrast, wavelet and EMD decomposition methods remain flexible and efficient for handling various noise types.

In addition to traditional methods like wavelet transforms, EMD, and Kalman filtering, newer approaches for ECG denoising offer distinct advantages but also present unique challenges. Sparse representation techniques effectively eliminate noise by exploiting signal sparsity. Selesnick et al. address waveform smoothness and peak underestimation issues, but their methods still struggle with baseline wander removal [136,152]. Wang et al. improve this by integrating LTI filtering, though their approach requires manual parameter adjustment [52].

Probabilistic models, such as the Bayesian framework introduced by Cuomo et al., show promising results, but their dependence on specific sensors limits clinical application [75,153]. SSA-based methods also demonstrate strong performance. Yang et al. offer guidance for window length selection *L*, while Mukhopadhyay et al. introduce a data-driven SSA approach that dynamically adapts *L* based on ECG morphology, reducing the need for manual tuning [22,134]. Mourad’s two-stage algorithm combines group sparsity and SSA to improve denoising, though parameter selection remains a challenge [154].

In recent years, real-time ECG signal denoising techniques have advanced alongside the development of mobile and wearable devices. For instance, Cuomo et al. propose a recursive filtering method for real-time ECG denoising with low computational cost, making it suitable for mobile health monitoring applications [75]. Similarly, Tripathi et al. develop an FD-based real-time ECG denoising method, utilizing fast Fourier transform to reduce computational complexity while efficiently suppressing various types of noise, making it ideal for real-time heart monitoring systems [155]. Yu et al. introduce a real-time wavelet thresholding method based on the autocorrelation function, which eliminates baseline drift, white noise, and electrode motion artifacts without complex parameter tuning, enhancing the visualization of denoised signals [58]. Additionally, Cuomo et al. enhance recursive filtering by optimizing the filter kernel for real-time noise removal, enabling local signal processing and visualization directly on mobile devices and wearable sensors [49].

**Figure 8:**
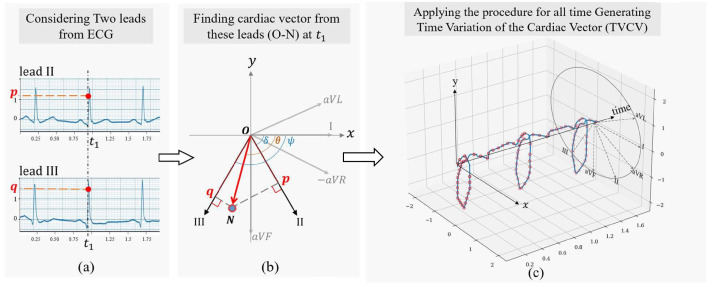
Vector-based post-processing method for improving ECG denoising techniques. (**a**) Leads II and III for a patient used to obtain the heart’s electrical vector. Using lead II (*p*) and lead III (*q*) values at $t_{1}$, we calculate the cardiac vector in the xy plane. (**b**) Perpendiculars from *p* and *q* to leads II and III axes intersect at *N*. Vector O-N represents the frontal plane cardiac vector at $t_{1}$. (**c**) Repeating this for a full cycle, we obtain the time variation of the cardiac vector 3D curve. Figure is modified from [156].

The aforementioned methods primarily address the denoising of a single ECG lead or channel, without accounting for the correlation or similarity between multiple leads or channels. Ghafari et al. propose a post-processing algorithm that calculates the direction and magnitude of the cardiac vector at each moment based on the electrical activity measured by limb lead signals [156]. This process produces the cardiac vector, as illustrated in Figure 8. By selecting the curve with the minimum denoising error, the algorithm reconstructs the denoised ECG signal. In addition to restoring the Einthoven relationship, this method effectively reduces residual noise and can enhance the performance of any denoising technique applied to limb lead signals.

A growing body of research explores deep neural networks for denoising ECG signals, with commonly employed models including GANs [157], DAEs [78], CNNs [158], and self-attention-based architectures [159]. Transformer-based convolutional networks further enhance this field by capturing complex dependencies in ECG data [160]. Wang et al. propose a supervised deep factor analysis-based algorithm for ECG denoising that demonstrates strong performance in handling complex noise and robustness across different conditions [80]. In parallel, Dias et al. develop a denoising technique based on GRUs that simplifies the training process by focusing on recurrent temporal dependencies. This approach maintains high accuracy, with an SNR improvement of 19.8 dB and PRD of 6.4%, effectively handling complex noise while reducing computational complexity [74]. However, although GRU-based models effectively reduce noise, they face challenges related to their limited interpretability.

To address this issue, hybrid methods combining deep learning with model-based approaches emerge as a promising direction. Hou et al. introduce a deep learning framework using sparse representation methods, enhancing noise suppression by leveraging the sparsity of ECG signals [161]. Kaergaard et al. integrate deep learning models with DWT, utilizing the time–frequency localization properties of DWT along with the feature extraction capabilities of neural networks [162]. Similarly, Xiong et al. propose a method that incorporates autoencoders and wavelet transforms with soft thresholding, optimizing noise suppression while preserving crucial ECG features [70].

**Table 5:** Summary of composite denoising on EC signal (A).

Methods	Database	Record	Evaluation Parameters
Schrodinger equation followed by a new wavelet [38]	MIT-BIH and experimental data	105, 234	${\mathrm{SNR}}_{\mathrm{imp}}$ = 20 dB (avg.) MSE = 0.014 (avg.) PRD = 0.014 (avg.)
Variable frequency complex demodulation (VFCDM) algorithm [59]	MIT-BIH and Wearable Armnand ECG Data and NSTDB	100, 101, 103, 105, 106, 115, 215 and 230	${\mathrm{SNR}}_{\mathrm{imp}}$ = 7.5015 dB, PRD = 7.44%, WEDD = 6.52% (for 15 dB I/P SNR)
Variable-frequency complex demodulation (VFCDM) algorithm [134]	Experimental data	-	Emax = 19.01%, Eavg = 7.88%, WEDD = 4.70%
Singular spectrum analysis and digital filtering [22]	MIT-BIH, NSTDB, PTBDB	-	${\mathrm{SNR}}_{\mathrm{imp}}$ = 41.96 dB, PRD = 5.71%, WEDD = 2.99% (for MIT-BIH)
Periodic non-local means filter [92]	MIT-BIH, Real noisy ECG signals	103, 105, 106, 109, 115, 121, 201, 203, 209, 231 and 233	${\mathrm{SNR}}_{\mathrm{imp}}$ = 8.855 dB, MSE = 0.0010, PRD > 10% (for 10 dB I/P SNR)
Bandpass filter and group sparsity and singular spectrum analysis [154]	Simulate ECG data	s20011, s20031, s20041, s20051, s20061, s20071, s20081, s20091 and s20101	${\mathrm{SNR}}_{\mathrm{imp}}$ = 10 dB (for 10 dB I/P SNR)
Adaptive dual-threshold filter and discrete wavelet transform [104]	MIT-BIH	100, 101, 103, 105, 115, 122, 124 and 231	${\mathrm{SNR}}_{\mathrm{imp}}$ = 7.143 dB, RMSE = 0.0042 (for 5 dB I/P SNR)
Discrete wavelet transform and non-local means (NLM) estimation [143]	MIT-BIH	100, 103, 105, 106, 115, 215	${\mathrm{SNR}}_{\mathrm{imp}}$ = 8.064 dB (for 10 dB I/P SNR), MSE = 0.0037, PRD = 5.5%
Dual-tree wavelet transform [71]	Simulated ECG data	203, 109, 119, 111 and 108	SNR = 30.493 dB (for 5 dB I/P SNR for record 108)
Biorthogonal wavelet transform and adaptive slope prediction-based threshold [72]	MIT-BIH, PTB, ST	80 + 365 records	SNR = 32.6568 dB, MSE = 0.0003
Biorthogonal wavelet transform and wavelet-Wiener filter thresholding [139]	QT database	46 records	Avg. SNR = 4.2568 dB, MSE = 0.008, Avg. PRD = 12.14% (10 dB) Avg. SNR = 4.5594 dB, Avg. MSE = 0.0023, Avg. PRD = 11.19% (5 dB)
Local means (LM) method [137]	MIT-BIH, ST	100, 103, 104, 105, 106, 115, 215	Avg. ${\mathrm{SNR}}_{\mathrm{imp}}$ = 10 dB, Avg. MSE = 0.0003, Avg. PRD < 12% (10 dB I/P SNR) Avg. ${\mathrm{SNR}}_{\mathrm{imp}}$ = 10 dB, Avg. MSE = 0.0003, Avg. PRD ≈ 15% (for 10 dB I/P SNR)
Riemann–Liouville fractional integral filtering and Savitzky–Golay filtering and empirical mode decomposition [76]	MIT-BIH	115	SNR = 7.5288 dB, MSE = 0.0027
EMD and non-local means [149]	MIT-BIH	215, 115, 106, 105, 104, 103, 100	Avg. ${\mathrm{SNR}}_{\mathrm{imp}}$ > 8 dB, Avg. MSE = 0.000472, Avg. PRD < 20% (for 10 dB I/P SNR)
EMD and adaptive switching mean filter (ASMF) [98]	MIT-BIH	100, 101, 103, 105, 115, 200, 215, and 230	${\mathrm{SNR}}_{\mathrm{imp}}$ > 8 dB, MSE ≈ 0.002, PRD < 15% (for 10 dB I/P SNR)
Eigenvalue decomposition of the Hankel matrix [77]	MIT-BIH	100, 101, 103, 105, 108, 109, 111, 112, 113, 115, 116, 117, 118, 121, 122, 123, 210, and 212	${\mathrm{SNR}}_{\mathrm{out}}$ = 12.89 dB, PRD ≈ 20% (for 5 dB I/P SNR and record 100)

**Table 6:** Summary of composite denoising on ECG signal (B).

Methods	Database	Record	Evaluation Parameters
Dual-tree complex wavelet transform and non-negative garrotte threshold function [141]	MIT-BIH	all 48 records	SNR = 58.23 dB, MSE = 0.0000000963, PRD = 0.001 (for record 100)
Cooperative filtering of similar segments and Savitzky–Golay and polynomial fitting [138]	MIT-BIH	100, 101, 103, 104, 105, 106, 113, 115, 200, 215, 230 and 231	${\mathrm{SNR}}_{\mathrm{imp}}$ ≥ 8 dB, MSE = 0.0010, PRD = 23.87% (for 5 dB I/P SNR and record 100)
Running denoising autoencoder [78]	Simulated ECG signals	-	${\mathrm{SNR}}_{\mathrm{imp}}$ ≈ 20 dB, MSE < 0.00005 (for 5 dB I/P SNR)
Non-local wavelet transform domain filtering [142]	MIT-BIH, PTB	100, 103, 104, 105, 106, 115 and 215, s0032, s0207, s0508, s0510, s0430, s0035, s0354, s0370, s0003, s0012, s0432, s0390	${\mathrm{SNR}}_{\mathrm{imp}}$ ≈ 6 dB, PRD ≈ 12%, MSE < 0.003 (for 20 dB I/P SNR and record 103), ${\mathrm{SNR}}_{\mathrm{imp}}$ = 19.18 dB, PRD = 19.1%, MSE = 0.001 (for record s0032)
DAE using the fully convolutional network (FCN) [158]	MIT-BIH	all 48 records	Avg. ${\mathrm{SNR}}_{\mathrm{imp}}$ > 8 dB, Avg. RMSE = 0.063, Avg.PRD = 19.68% (for −1 dB I/P SNR)
Stationary wavelet total variation algorithm [102]	MIT-BIT	100, 103, 105, 113, 115, 117, 119, 122, 200, 215, 213, 230, 231 and 234	SNR = 25.44 dB, RMSE = 0.3940, PRD = 40% (for 5 dB I/P SNR at record 231)
Fractional Stockwell transform (FrST) [79]	MIT-BIT, ST	100, 101, 102, 103, 113, 201, 207, 217, 231, e0103, e0104, e0105 and e0106	${\mathrm{SNR}}_{\mathrm{imp}}$ = 19.0699 dB, RMSE = 0.0807, PRD ≈ 8% (for 15 dB I/P SNR and record 100), ${\mathrm{SNR}}_{\mathrm{imp}}$ = 24.6292 dB, RMSE = 0.0469 (for 15 dB I/P SNR and record e0103)
Ensemble empirical mode decomposition and genetic-algorithm-based thresholding technique [148]	MIT-BIH	-	${\mathrm{SNR}}_{\mathrm{imp}}$> 5 dB, MSE < 0.05, PRD < 20% (for 10 dB I/P SNR)
Real-time accurate thresholding method and discrete wavelet transform [58]	MIT-BIH	233	${\mathrm{SNR}}_{\mathrm{imp}}$ = 6.0286 dB, RMSE = 0.2705
Particle swarm optimization and wavelet transform [117]	MIT-BIH	100	SNR = 14.5189 dB, MSE = 0.143, RMSE = 0.1103, PRD = 20.1725% (for 5 dB I/P SNR)
U-Net [73]	PTB-XL, CPSC2018	Manually label	SNR = 20.60 dB, RMSE = 0.0111 (for 20 dB I/P SNR)
Transformer encoder [163]	QT database	-	SNR = 13.60 dB, RMSE = 0.06, PRD = 22.85% (for 0–6 dB I/P SNR)
Sparse coding and Kalman filter [145]	QT database	-	${\mathrm{SNR}}_{\mathrm{imp}}$ = 18.6 dB, MAE = 0.026, (for −5 dB I/P SNR)
Bidirectional gated recurrent units [74]	Experimental data collected using wearable sensors	-	${\mathrm{SNR}}_{\mathrm{imp}}$ = 18.9 dB, RMSE = 0.029, PRD = 6.4%
Transformer and convolutional network [160]	MIT-BIH	all 48 records	${\mathrm{SNR}}_{\mathrm{out}}$ = 8.49 dB, MSE = 0.035, (for 0 dB I/P SNR)

In light of interpretability concerns, the development of explainable neural networks is gaining momentum. Attention visualization techniques, for example, allow researchers to determine whether the model focuses on relevant signal characteristics or noise during denoising. These advancements suggest that integrating attention mechanisms or other explainable AI techniques not only enhances denoising performance but also offers valuable insights into how the model processes signals, which could be critical in clinical applications. Moreover, multi-task learning is likely to become a key focus in ECG signal analysis. With the increasing availability of medical data and advancements in deep learning, multi-task learning can efficiently integrate denoising, signal quality assessment (SQA), and diagnosis tasks, improving overall signal analysis. Recent studies explore multi-task learning models that combine ECG denoising with SQA and disease diagnosis, offering a more holistic approach to ECG processing [164,165]. This approach not only enhances processing efficiency but also opens pathways for personalized and intelligent healthcare systems.

## 5. Noises in MCG Signal

The MCG signal is extremely weak, with amplitudes typically ranging from 10 to 100 pT, which is one millionth of the Earth’s magnetic field intensity [166,167]. To capture such weak MCG signals, magnetometers are usually highly sensitive. Quantum sensing has been applied in various fields, including inertial and magnetic field measurements [168,169,170,171,172,173]. Currently, the primary commercial device for MCG is the superconducting quantum interference device (SQUID) magnetometer. The SQUID is an efficient magnetic flux-to-voltage converter based on flux quantization and the Josephson effect, capable of detecting weak magnetic fields [174]. However, SQUIDs must operate at ultra-low temperatures and require cryogenic cooling with liquid helium, which is costly and limits their clinical deployment due to poor flexibility. In recent years, high-sensitivity OPMs, such as spin exchange relaxation-free (SERF) OPMs, have become a research focus. OPMs eliminate the need for cryogenic cooling with liquid helium, significantly reducing device size and enabling system miniaturization [175]. They also enhance the flexibility of arranging multi-channel measurement sensor arrays. Additionally, tunneling magnetoresistance (TMR) sensors have made significant progress in the field of MCG measurement in recent years, eliminating the requirement for magnetic shielding devices [176]. However, TMR sensors may exhibit drawbacks such as lower sensitivity and resolution, susceptibility to environmental interference, and manufacturing complexity. When selecting an appropriate MCG measurement technology, evaluation and selection should be based on specific application requirements and technical considerations.

Due to the high sensitivity requirements of magnetometers, MCG signals contain complex noise components. Noise reduction in the MCG signal is primarily achieved through three methods: 1. passive or active magnetic shielding [177]: passive magnetic shielding involves the use of shielded rooms or shielded cylinders made of high-permeability materials, while active magnetic shielding compensates for the three-axis magnetic field by installing demagnetization coils inside shielded rooms or magnetometers. 2. Constructing gradient magnetometers for differential noise cancellation [178]. 3. Software processing [179,180,181]: utilizing signal processing techniques to remove noise and improve the signal-to-noise ratio. In most cases, a combination of these methods, or at least two of them, is required to extract useful signals and achieve satisfactory signal-to-noise ratios [26].

**Figure 9:**
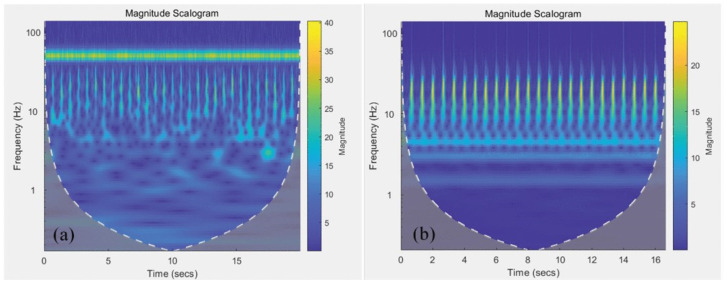
Time–frequency graph of (**a**) original and (**b**) processed MCG signals [177].

MCG measurements are typically recorded within a magnetically shielded room (MSR), which can shield most of the geomagnetic interference. However, different shielding techniques still result in residual levels of geomagnetic noise [182]. Additionally, the measured MCG signal inevitably suffers from various types of artifacts. The time–frequency characteristics of the MCG signal are illustrated in Figure 9. The original signal is heavily affected by PLI noise. Apart from PLI noise, much of the noise originates from low-frequency components (<10 Hz). Low-frequency noise primarily includes signals related to subject chest movements relative to the sensor induced by respiration, thermal noise due to temperature instability, baseline drift caused by fiber optic jitter, and electronic 1/f noise [177]. In addition to the persistent noise sources mentioned above, there are also occasional artifacts, for instance, EMG caused by subject movement and metallic artifacts. Metallic materials possess magnetism or conductivity, leading to local magnetic field irregularities that interfere with accurate MCG measurements. This interference can lead to erroneous signal patterns or signal loss in the measurement results, affecting the correct assessment of cardiac activity. Therefore, before conducting MCG measurements, subjects should remove all metal items from their bodies. However, if subjects have implanted metallic objects (such as surgical clips, prosthetics, dental fillings, etc.), the potential impact of metallic artifacts should be evaluated and minimized as much as possible.

## 6. Pre-Processing in MCG Signal

**Figure 10:**
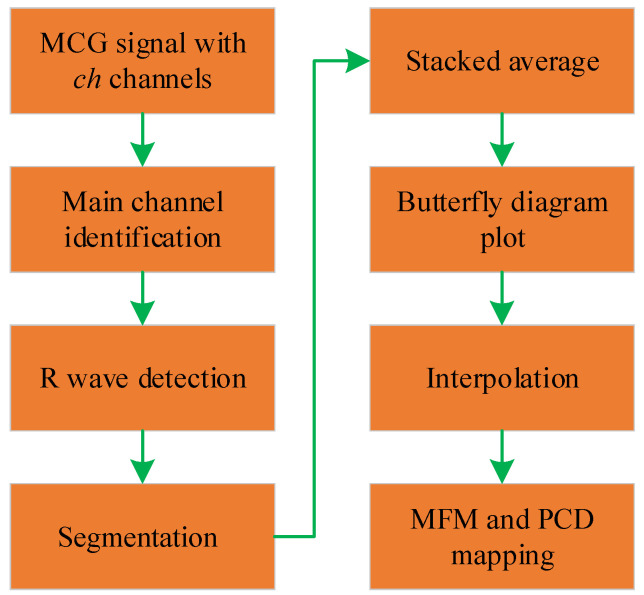
Flowchart of the ensemble averaging algorithm.

Simple preprocessing methods may result in incomplete noise removal or excessive filtering, leading to changes or loss of characteristic waveforms in the MCG signal and hindering subsequent MCG signal analysis. Therefore, it is necessary to find an appropriate method to remove noise without altering the characteristics of the MCG signal. To mitigate the impact of various types of noise on MCG signals, researchers have proposed various denoising strategies. Due to the periodicity of the MCG signal, analyzing the information of a single cycle is sufficient to capture most of the cardiac information, making the stack averaging algorithm widely used in the preprocessing of MCG signals. The flowchart of the stack averaging algorithm is shown in Figure 10.

**Figure 11:**
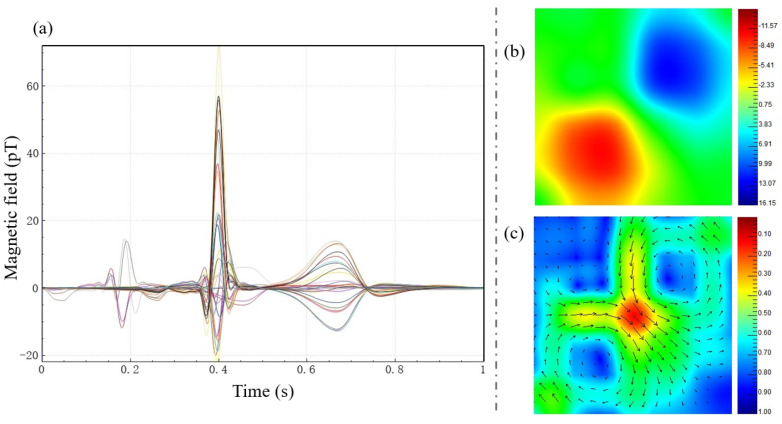
(**a**) Butterfly plot, (**b**) MFM, and (**c**) MCD maps of MCG signals in an adult.

Firstly, in ch channels, a main channel is selected, and the R-wave positions and the number of R waves *K* in the main channel are identified. Next, each channel will be segmented into *K* single-cycle signals, with the R-wave position as the reference. Then, each of the *K* single-cycle signals for each channel will be separately averaged to obtain ch stacked average signals. Finally, the stacked average signals from ch channels are plotted on the same graph to form a butterfly plot. The butterfly plot is a common visualization method for MCG signals, providing an overview of the waveform characteristics of multi-channel MCG signals. In addition to butterfly plots, magnetic field maps (MFMs) and pseudocurrent density (PCD) maps are commonly used as two-dimensional visual representations of MCG signals and have been applied in medical diagnostics [183]. Figure 11a–c depict the butterfly plot and their corresponding MFM and PCD map for a normal individual. In the MFM at the peak of the T wave in healthy adults, there should exist a pair of symmetric dipoles.

The MFM is a contour plot formed by connecting measurement points with the same magnitude of MCG signals obtained at the same moment. It is filled with colors, where red represents the maximum positive magnetic field and blue represents the maximum negative magnetic field. The formula for calculating the current vector in the PCD map is as follows [184,185]:

(2) $$\begin{array}{c} \vec{c}=\frac{\partial B_{z}}{\partial y}\vec{e_{x}}-\frac{\partial B_{z}}{\partial x}\vec{e_{y}} \end{array}$$

where the *x*-axis points from the heart to the head, representing the vertical direction along the body’s longitudinal axis (bottom to top); the *y*-axis extends from the right shoulder to the left shoulder, representing the horizontal direction along the body’s transverse axis (right to left); and the *z*-axis extends from the chest to the back, representing the depth along the body’s anteroposterior axis (front to back). $\vec{c}$ represents the current vector on the measurement plane, $B_{z}$ is the normal component of the magnetic flux density of the heart collected by the magnetometer, and $\vec{e_{x}}$ and $\vec{e_{y}}$ are the unit directional vectors in the *x* and *y* directions on the measurement plane. Therefore, their magnitude and phase are, respectively,

(3) $$\begin{array}{c} \left|c\right|=\sqrt{{\left(\frac{\partial B_{z}}{\partial y}\right)}^{2}+{\left(\frac{\partial B_{z}}{\partial x}\right)}^{2}} \end{array}$$

(4) $$\begin{array}{c} \theta =\arctan\left(-\frac{\partial B_{z}}{\partial x}/\frac{\partial B_{z}}{\partial y}\right) \end{array}$$

In practical calculations, differences are used in place of partial derivatives, with $\frac{\Delta B_{z}}{\Delta x}$ and $\frac{\Delta B_{z}}{\Delta y}$ substituting for $\frac{\partial B_{z}}{\partial x}$ and $\frac{\partial B_{z}}{\partial y}$, respectively. Here, Δx and Δy represent the spacing between adjacent data points in the *x* and *y* directions. To improve accuracy, the central difference method is used for positions in the middle, while a first-order forward difference method is used for points at the boundaries, as shown in (5) and (6), respectively:

(5) $$\begin{array}{c} \frac{\partial B_{z(i)}}{\partial x}=\frac{B_{z(i+1)}-B_{z(i-1)}}{2\Delta x},\\ \frac{\partial B_{z(i)}}{\partial y}=\frac{B_{z(i+1)}-B_{z(i-1)}}{2\Delta y} \end{array}$$

(6) $$\begin{array}{c} \frac{\partial B_{z(i)}}{\partial x}=\frac{B_{z(i+1)}-B_{z(i)}}{\Delta x},\\ \frac{\partial B_{z(i)}}{\partial y}=\frac{B_{z(i+1)}-B_{z(i)}}{\Delta y} \end{array}$$

The current vectors at various positions are plotted in the form of arrow diagrams, forming the PCD map. The PCD map is considered as a two-dimensional projection of the three-dimensional current distribution and can be used to estimate the underlying current sources and their propagation processes.

The stack averaging algorithm effectively removes random noise, significantly enhancing the quality of MCG signals. However, stack averaging is not applicable to all types of noise. It has limited effectiveness against non-random, periodic noises commonly found in MCG signals, such as PLI, BW, or EMG. These types of noise may remain relatively stable across multiple recordings, rendering simple averaging ineffective for their elimination. Additionally, an important characteristic of MCG signals is heart rate variability, which refers to small variations in the intervals between heartbeats. During stack averaging, if there are slight differences in the heartbeat cycle, the averaging process may eliminate these physiologically significant variations, thereby impacting the assessment of cardiac function. Furthermore, the stack averaging denoising method is not universally applicable to the diagnosis of all types of cardiac diseases. If abnormal heartbeats (such as premature beats) are present in the acquisition sequence, stack averaging may erroneously treat these important but irregular signals as noise and attenuate them, thereby affecting the accurate diagnosis of cardiac pathological conditions. Therefore, although stack averaging is a method for attenuating random noise, in practice, it often requires the integration of additional denoising techniques tailored to the characteristics of MCG signals.

The waveform characteristics of MCG are similar to those of ECG; thus, denoising methods applied to ECG signals are widely used in MCG denoising, as shown in Table 7. Traditional digital filtering methods have been utilized for MCG denoising; however, due to the similarity between certain noise spectra and MCG signals, this approach may lead to signal distortion [186,187]. High-order IIR filters have become a choice for addressing PLI and BW issues in MCG denoising. Although this method achieves efficient filtering at low computational cost, it sacrifices phase linearity [188]. The introduction of wavelet transform provides a new approach for MCG signal processing [189]. However, the selection of wavelet bases is crucial for denoising effectiveness and is subjective. Bing et al. apply a heart Fourier wavelet denoising method based on sparse representation to process MCG signals. This algorithm effectively eliminates noise in MCG signals, improving the signal-to-noise ratio. However, similar to the drawbacks of wavelet denoising, the denoising effectiveness highly depends on the choice of wavelet bases and requires the establishment of an overcomplete dictionary [180].

**Figure 12:**
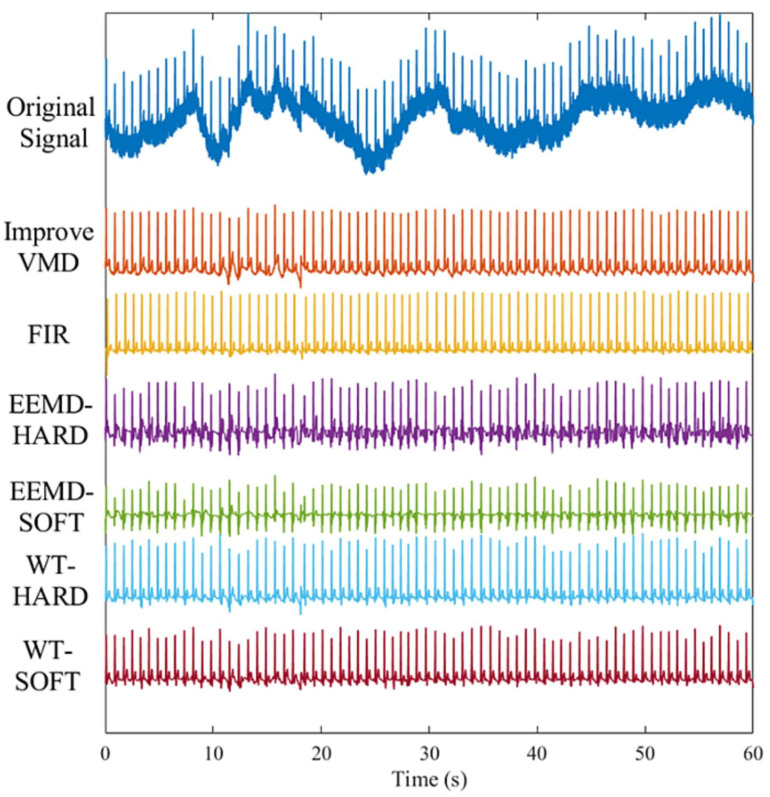
Comparison of the effect of different algorithms on real MCG signal after processing [178].

EMD has been widely used for MCG denoising due to its ability to decompose signals without the need for predefined bases. Mariyappa et al. utilize EEMD to eliminate high-frequency random noise, PLI, and low-frequency BW in measured MCG data [27]. Although EEMD reduces mode mixing by adding random noise multiple times and averaging, it may still leave residual noise in the decomposed signal, affecting the accuracy of signal reconstruction. CEEMDAN further enhances EEMD by adaptively adding white noise with different amplitudes and distribution characteristics to improve decomposition stability and accuracy, resulting in purer reconstructed signals. Lu et al. apply CEEMDAN to MCG signal denoising; however, this method is computationally complex [190]. MCG signals possess high spatiotemporal resolution, and signals from different locations naturally carry distinct characteristics. Multi-channel MCG acquisition allows for a comprehensive capture of the cardiac electrophysiological magnetic field. Blind source separation methods are widely employed in multi-channel signal processing. This spatial filtering technique effectively suppresses environmental noise and non-cardiac source signals, enhancing the signal strength in regions of interest and thereby improving the signal-to-noise ratio. Yang et al. combine EMD and ICA methods for MCG signal denoising, yielding significantly improved MCG waveforms [26]. Mariyappa et al. employ a combination of EEMD and ICA methods for denoising multi-channel MCG data. The PCD images obtained from EEMD-ICA offer clear and well-resolved information related to the activation centers during ventricular repolarization in normal subjects [191]. Chen et al. propose a denoising algorithm for MCG signals in an unshielded room environment. This method combines the whale optimization algorithm with the VMD algorithm to achieve blind source separation of MCG signals in an unshielded environment. The proposed method is compared with common MCG signal processing methods, as shown in Figure 12. The optimized VMD method exhibits superior noise reduction capability and baseline drift removal effects. However, this algorithm is complex, relatively slow, and requires the manual setting of VMD parameters [178].

The adaptive filtering method effectively eliminates environmental noise [192]. It employs a set of independent reference channels dedicated to measuring environmental noise and measures the channels of interest affected by noise. Patel et al. propose an effective adaptive method to suppress respiration-related baseline drift artifacts. This method utilizes temperature sensors to monitor respiratory signals and employs regression techniques to remove respiratory artifacts from contaminated MCG signals [193]. However, the adaptive method requires the introduction of additional sensors as reference signals.

Some studies focus on utilizing AI techniques to eliminate 1/f noise from MCG signals, achieving notable effectiveness. Nevertheless, this approach demands a substantial amount of data for training and requires extensive training time and computational resources [181,194,195]. Sengottuvel et al. propose a correlation-based beat-by-beat approach and principal component analysis to eliminate drifts and noise from implanted devices. This approach is suitable for denoising single-channel and multi-channel MCG data. However, it relies on the accurate detection of R waves. When noise overwhelms the R-wave detection, the effectiveness of this method diminishes [25].

**Table 7:** Summary of denoising methods on MCG signal.

Methods	MCG Acquiring Device	Noise Source	Evaluation Parameters
Variational mode decomposition (VMD) and whale optimization algorithm (WOA) [178]	Simulated MCG signal and actual MCG signals collected by SQUID gradiometers	Baseline drift noise, industrial frequency noise, and Gaussian white noise	${\mathrm{SNR}}_{\mathrm{imp}}$ = 11.7892 dB, PRD = 0.5841, CC = 0.7218 (for 0 dB I/P SNR)
Independent component analysis (ICA) and ensemble empirical mode decomposition (EEMD) [191]	Actual MCG signals collected by a 37- channel SQUID	High-frequency baseline drifts, low-frequency baseline drift, breathing artifact, 50 Hz PLI, high-frequency random noise, etc.	${\mathrm{SNR}}_{\mathrm{imp}}$ ≈ 11.7892 dB
Convolutional neural network (CNN) [181]	Simulated MCG signal	1/f noise	RMSE ≈ 0.03 (avg.)
Fourier wavelet denoising and sparse representation [180]	Simulated noise	High-frequency noise, power frequency noise, and low-frequency noise	SNR = 10.5521 dB, MSE = 0.0199 (for high-frequency noise) SNR = 17.5935 dB, MSE = 0.0035 (for power frequency noise) SNR = 10.4488 dB, MSE = 0.0232 (for low-frequency noise)
Correlation-based beat-by-beat approach and principal component analysis (PCA) [25]	Actual MCG signals collected by a 37- channel SQUID	Subjects with implanted devices	${\mathrm{SNR}}_{\mathrm{imp}}$ = 30 dB
Signal space separation (SSS) and projection operation [179]	Actual MCG signals collected by 48-channel tunneling magnetoresistance (TMR) sensors	Environmental magnetic sensor noise	Reduces the environmental magnetic noise by −73 dB and the sensor noise by about −23 dB
Independent component analysis (ICA) and EMD [26]	Actual MCG signals collected by an SERF atomic magnetometer array	Environmental noise, baseline drift, respiratory interference, and power line noise	Obvious characteristics of P wave, QRS wave, and T wave

## 7. Performance Evaluation

Various evaluation parameters are used to assess ECG/MCG denoising algorithms, forming an integral part of signal denoising analysis. These parameters not only evaluate the algorithms but also serve as a medium for comparing various proposed algorithms. Since ECG/MCG signals contain rich physiological information, a single evaluation parameter is insufficient to comprehensively reflect the overall performance of denoising algorithms in preserving signal characteristics and reducing noise interference. Therefore, in the literature, multiple parameters are often chosen to comprehensively assess algorithm performance from different perspectives, providing a more comprehensive evaluation. In this section, evaluation parameters are discussed in detail. In the following equations, Y(T), X(T), and $\tilde{Y}\left(T\right)$ represent a clean noiseless ECG signal, original signal, and denoised signal, respectively. n is the length of the ECG/MCG signal.

Input-signal-to-noise ratio (${\mathrm{SNR}}_{\mathrm{in}}$):

(7) $$\begin{array}{c} {\mathrm{SNR}}_{\mathrm{in}}=10\log_{10}\left(\frac{\sum_{T=1}^{n}{\left(Y\left(T\right)\right)}^{2}}{\sum_{T=1}^{n}{\left(X\left(T\right)-Y\left(T\right)\right)}^{2}}\right) \end{array}$$

Output-signal-to-noise ratio (${\mathrm{SNR}}_{\mathrm{out}}$):

(8) $$\begin{array}{c} {\mathrm{SNR}}_{\mathrm{out}}=10\log_{10}\left(\frac{\sum_{T=1}^{n}{\left(Y\left(T\right)\right)}^{2}}{\sum_{T=1}^{n}{\left(\tilde{Y}\left(T\right)-Y\left(T\right)\right)}^{2}}\right) \end{array}$$

Signal-to-noise ratio improvement (${\mathrm{SNR}}_{\mathrm{imp}}$):

(9) $$\begin{array}{c} {\mathrm{SNR}}_{\mathrm{imp}}=10\log_{10}\left(\frac{\sum_{T=1}^{n}{\left(X\left(T\right)-Y\left(T\right)\right)}^{2}}{\sum_{T=1}^{n}{\left(\tilde{Y}\left(T\right)-Y\left(T\right)\right)}^{2}}\right) \end{array}$$

Mean squared error (MSE):

(10) $$\begin{array}{c} \mathrm{MSE}=\frac{1}{n}\sum_{T=1}^{n}{\left(\tilde{Y}\left(T\right)-Y\left(T\right)\right)}^{2} \end{array}$$

Normalized form of MSE (NMSE) [196]:

(11) $$\begin{array}{c} \mathrm{NMSE}=\frac{\sum_{T=1}^{n}{\left(\tilde{Y}\left(T\right)-Y\left(T\right)\right)}^{2}}{\sum_{T=1}^{n}{\left(Y\left(T\right)\right)}^{2}} \end{array}$$

Correlation coefficients (CCs):

(12) $$\begin{array}{c} \mathrm{CC}=\frac{\sum_{T=1}^{n}Y\left(T\right)\tilde{Y}\left(T\right)}{\sqrt{\sum_{T=1}^{n}Y^{2}\left(T\right)\sum_{T=1}^{n}{\tilde{Y}}^{2}\left(T\right)}} \end{array}$$

The larger the correlation coefficient, the better the denoising effect of the algorithm.

Percentage root-mean-square difference (PRD):

(13) $$\begin{array}{c} \mathrm{PRD}=100\sqrt{\frac{\sum_{T=1}^{n}\left({\left(\tilde{Y}\left(T\right)-Y\left(T\right)\right)}^{2}\right)}{\sum_{T=1}^{n}Y^{2}\left(T\right)}} \end{array}$$

Root-mean-square error (RMSE):

(14) $$\begin{array}{c} \mathrm{RSME}=\sqrt{\frac{1}{n}\sum_{T=1}^{n}{\left(\tilde{Y}\left(T\right)-Y\left(T\right)\right)}^{2}} \end{array}$$

Power spectral density (PSD) analysis and mean frequency (MF) can provide a deeper evaluation of the denoising method’s performance. PSD indicates how the power of a signal is spread across different frequencies [197]. PSD shows whether the signal is strong or weak at each frequency change. If we integrate the PSD within a specific frequency range, we can calculate the energy of that frequency. This can be carried out using FFT or autocorrelation functions.

(15) $$\begin{array}{c} MF=\sum_{j=1}^{b}f_{j}P_{j}/\sum_{j=1}^{b}P_{j}, \end{array}$$

where $f_{j}$ is the frequency value of the ECG power spectrum at the frequency bin *j* and $P_{j}$ is the power spectrum of the ECG at the frequency bin *j*. *b* is the length of the frequency bin.

## 8. Discussion and Challenges

ECG and MCG detection hold significant importance in the field of cardiac diseases. ECG, as a conventional electrocardiographic method, records changes in the timing and intensity of cardiac electrical activity, aiding in the diagnosis of cardiac conditions like arrhythmias and myocardial ischemia. On the other hand, MCG emerges as a novel cardiac magnetic field detection method, characterized by its non-invasiveness and high precision. MCG can directly detect the magnetic fields generated by the heart, unaffected by tissues.

Multiple studies indicate that MCG and ECG provide complementary information. However, ongoing debate exists regarding whether the information detected by MCG and ECG pertains to the source level (internal heart activity) or the sensor level. No unified conclusion is reached. The reasons for this controversy are multifaceted. Firstly, the heart is a complex bioelectric system with electrical activity involving various types and directions of currents. Therefore, the generation and detection of MCG and ECG signals involve complex biophysical processes, with many potential influencing factors and sources of interference. Secondly, there are physiological and anatomical differences among individuals, leading to variations in the characteristics of cardiac electrical activity and signal features. Additionally, different pathological states and cardiovascular diseases can also affect the characteristics of MCG and ECG signals, contributing to heterogeneity in research. Considering these factors, a deeper understanding of the relationship between MCG and ECG signals requires further research and exploration. Future studies should incorporate more refined electrophysiological models, advanced signal processing techniques, and stricter experimental designs to elucidate the relationship between MCG and ECG signals, thus providing more accurate information for clinical applications.

Denoising ECG and MCG signals is crucial. These signals share some commonalities and differences in their denoising processes. Both ECG and MCG are subject to interference from various sources, including biological signals, equipment disturbances, and environmental noise. Therefore, it is essential to apply appropriate technical methods to suppress these noises for both types of signals. However, due to differences in signal characteristics and measurement methods, the denoising strategies and techniques for ECG and MCG signals can vary. Denoising MCG signals poses greater challenges compared to ECG signals. Firstly, the measurement of MCG involves specialized equipment, such as SQUIDs or OPMs, to detect the weak magnetic fields generated by the heart. This technique is susceptible to interference from environmental magnetic fields, electromagnetic noise from equipment, and the magnetic properties of the subject, making the sources of noise more complex. Secondly, MCG signals are extremely weak and have higher spatial resolution, making them more sensitive to noise. Any minor variations from the equipment, environment, or subject can significantly impact MCG signals, increasing the difficulty of denoising. Furthermore, the complexity and dynamic nature of MCG signals add to the challenge. MCG signals reflect the spatial distribution and variations in cardiac electrical activity, involving multiple types and directions of currents. Accurately identifying and removing noise from such multi-source, multi-directional signals is a formidable task.

The high cost of MCG acquisition equipment has led to limited research in this area. Currently, there is a lack of standard MCG databases, making it difficult to evaluate the effectiveness of denoising algorithms. Without an accepted “clean” MCG signal for reference, commonly used evaluation metrics cannot be employed to assess denoising performance. To address this issue, establishing and sharing standardized MCG databases or exploring alternative evaluation metrics is essential. One promising approach is the use of two-dimensional MCG images as an evaluation metric for denoising algorithms. MCG imaging provides a visual representation of the spatial distribution and variations in cardiac electrical activity, making it a valuable tool for assessing the effectiveness of denoising algorithms. An effective denoising algorithm should reduce background noise while preserving the primary features and structures of cardiac activity. By comparing MCG images before and after denoising, it is possible to evaluate the improvements in signal quality and structure. This method offers both a visual assessment and quantitative analysis, aiding in the evaluation of the algorithm’s effectiveness and accuracy in improving signal quality.

This review classifies ECG denoising techniques based on noise types, aligning more closely with real-world applications. It enables researchers to directly select the most suitable method for removing specific types of noise, conserving computational resources and improving signal processing efficiency and diagnostic accuracy. Different types of noise affect key ECG waveform features in different ways, impacting the diagnosis of certain diseases. For example, BW affects the accuracy of ST segments, T waves, and P waves, which can interfere with the detection of myocardial ischemia and infarction. PLI’s periodic high-frequency interference may obscure the detection of arrhythmias such as atrial fibrillation or ventricular tachycardia. Randomly occurring EMG noise is especially disruptive to the detection of transient arrhythmias like premature ventricular contractions. By categorizing denoising techniques in this way, researchers and clinicians can quickly select targeted approaches, optimizing noise removal, reducing misdiagnosis, and fostering the development of new technologies.

The development of deep learning techniques demonstrates superior capabilities in capturing spatiotemporal features, allowing for the effective separation of signal and noise across various temporal and spatial scales. With the incorporation of advanced methods such as attention mechanisms and self-supervised learning, deep learning shows enhanced adaptability and robustness, performing well in diverse data environments and applications. Consequently, deep-learning-based denoising techniques are expected to significantly improve signal processing accuracy and efficiency in this field. However, several challenges persist. First, deep learning models require substantial computational resources, which can introduce delays in real-time medical applications, thereby affecting the timeliness of signal processing and diagnosis. Second, the complexity of data annotation poses a challenge, as accurately labeling noise and meaningful signal segments in medical data is time-consuming and labor-intensive. This lack of sufficient annotations limits the effectiveness of model training. Finally, model interpretability is crucial for clinical applications, as healthcare professionals need to understand the rationale behind denoising decisions. Deep learning models, often viewed as “black boxes”, lack transparency, and while attention mechanisms offer partial solutions, current methods do not fully meet the demands of the medical field.

These challenges suggest that future research should focus on model simplification, automation of data annotation, and improving interpretability. Integrating denoising with tasks such as signal quality assessment and disease diagnosis through multi-task learning improves model performance by sharing representations across related tasks. This enables the model to both remove noise and assess signal quality, helping it to focus on preserving clinically relevant features. Additionally, combining disease diagnosis ensures that the denoising process is optimized to enhance diagnostic accuracy, leading to better generalization across diverse patient data. Moreover, multi-task learning reduces computational costs by training a single model for multiple tasks, making it suitable for real-time applications. This integration enhances the practicality of deep learning methods in intelligent diagnostic systems, enabling more accurate and efficient real-time signal processing and analysis.

This review still has several limitations. First, despite the numerous denoising techniques proposed by researchers, quantitatively comparing and evaluating these methods remains challenging. A major obstacle is the lack of standardized benchmarks: many techniques have not been evaluated using standard databases, and even when the same dataset is used, different studies often rely on varying recordings or signal-to-noise ratios. Furthermore, the sources of added noise are inconsistent—some studies use real noise extracted from databases, while others employ simulated noise, complicating direct comparisons. In addition, the inconsistency in evaluation metrics further complicates the assessment of these methods. To address this, establishing unified standards and benchmarks is essential for facilitating the meaningful comparison of different denoising techniques. Second, most studies evaluating ECG denoising methods focus on time-domain metrics, which can result in incomplete assessments. ECG signals possess not only rich time-domain features but also critical frequency-domain characteristics, such as frequency components and distributions. By limiting evaluation metrics to the time domain, these studies fail to fully assess how denoising algorithms affect the frequency-domain properties of the signals, potentially overlooking the removal or preservation of important noise components. Moreover, in clinical applications, evaluating denoising techniques should not only focus on denoising quality but also consider factors like algorithm speed, memory usage, and real-time performance, all of which are critical for practical implementation. However, few studies have provided comparative analyses of these factors, with only a limited number of papers discussing the real-time capabilities of the proposed methods. Future research needs to address these aspects, as they are essential for the practical application of denoising algorithms in real-world clinical settings.

## 9. Conclusions

This review presents a comprehensive examination of ECG and MCG signal preprocessing and denoising techniques, making significant contributions to the field of biomedical signal processing. One of the major strengths of this review is that it is the first to categorize and compare ECG denoising methods based on noise types. This approach is more aligned with real-world applications, enabling researchers to directly select the most appropriate method for removing specific types of noise. This can significantly improve processing efficiency and guide method selection based on the types of cardiac conditions being diagnosed, as different noise types affect critical ECG signal features differently. For MCG signals, this review addresses a notable gap in the literature by providing a detailed discussion of the unique noise characteristics and summarizing existing preprocessing techniques. By doing so, it contributes to advancing the clinical application of MCG in cardiovascular diagnosis, especially in combination with ECG, emphasizing the complementary nature of these two signal types.

We also discussed the future of denoising technologies, including the potential of deep neural networks. These methods show promise but face challenges such as high computational costs, the complexity of data labeling, and the need for better model interpretability. By integrating denoising with tasks like signal quality assessment and disease diagnosis through multi-task learning, there is potential to enhance model generalization, improve efficiency, and broaden the practical applicability of these methods in real-time and intelligent diagnostic systems.

However, several limitations remain. While some technologies have been applied to wearable devices for continuous heart monitoring, their accuracy is still limited. Additionally, most current research focuses on improving algorithmic accuracy and complexity but neglects real-time performance and hardware limitations. Future work should focus on optimizing algorithms for real-time use while maintaining high accuracy, exploring lightweight model designs, hardware acceleration, and multi-task learning. Lastly, the establishment of standardized experimental reporting practices is needed to facilitate the quantitative comparison of denoising methods, which will accelerate their clinical adoption.

## Figures and Tables

**Table 1:** Summary of BW denoising on ECG signal (A).

Methods	Database	Record	Evaluation Parameters
Variable-frequency complex demodulation (VFCDM) algorithm [59]	MIT-BIH and wearable armband ECG data and NSTDB	100, 101, 103, 105, 106, 115, 215, 230	${\mathrm{SNR}}_{\mathrm{imp}}$ = 4.17 dB, PRD = 5.79%, WEDD = 4.82% (for 20 dB I/P SNR)
Singular spectrum analysis and digital filtering [22]	MIT-BIH	115	${\mathrm{SNR}}_{\mathrm{imp}}$ = 29.19 dB, MSE = 0.000307
Fourier decomposition method (FDM) [40]	MIT-BIH	100, 101, 103, 105, 109, 111, 112, 113, 115, 116, 117, 118, 122, 123, 210, 212	${\mathrm{SNR}}_{\mathrm{out}}$ = 23.0 dB, PRD > 5% (for 5 dB I/P SNR and record 100)
Denoising autoencoder (DAE) [45]	1st China Physiological Signal Challenge 2018 (CPSC)	-	${\mathrm{SNR}}_{\mathrm{imp}}$ = 13.50 dB (for σ=0.06) ${\mathrm{SNR}}_{\mathrm{imp}}$ = 20.54 dB (for σ=0.14) ${\mathrm{SNR}}_{\mathrm{imp}}$ = 23.78 dB (for σ=0.22) ${\mathrm{SNR}}_{\mathrm{imp}}$ = 25.92 dB (for σ=0.30)
Linear time-invariant filtering and sparse optimization [51]	MIT-BIH	103, 105, 213	${\mathrm{SNR}}_{\mathrm{out}}$ = 17.87 dB, MSE = 0.003 (for 5 dB I/P SNR and record 103)
Recursive filtering [75]	Long-term ST	s20011, s20021, s20031, s20041, s20051, s20061, s20071, s20081, s20091 and s20101	SNR = 19.12 dB (s20011)
Dual-tree wavelet transform [71]	Simulated ECG data	-	${\mathrm{SNR}}_{\mathrm{imp}}$ = 15.24564 dB, MSE = 0.00044 (for 5 dB I/P SNR)
Biorthogonal wavelet transform and Adaptive slope prediction-based threshold [72]	MIT-BIH, BIDMC, PTB, ST	16 records 15 records 80 + 365 records 17 records	SNR = 28.3821 dB, MSE = 0.0029
Real-time accurate thresholding method and discrete wavelet transform [58]	MIT-BIH	233	${\mathrm{SNR}}_{\mathrm{imp}}$ = 3.03255 dB, RMSE = 0.24123
Recursive filtering [49]	Long-term ST	s20011, s20051, s20061, s20071, s20081 and s20102	SNR = 14.39 dB (s20012)
Riemann–Liouville fractional integral filtering and Savitzky–Golay (SG) filtering and EMD [76]	MIT-BIH	115	SNR = 7.6487 dB, MSE = 0.0026
Eigenvalue decomposition of the Hankel matrix [77]	MIT-BIH	100, 101, 103, 105, 108, 109,111, 112, 113, 115, 116, 117, 118, 121, 122, 123, 210, 211, 212	${\mathrm{SNR}}_{\mathrm{out}}$ = 8.39 dB, PRD ≈ 30% (for 5 dB I/P SNR and record 100)
Running denoising autoencoder [78]	Simulated ECG signals	-	${\mathrm{SNR}}_{\mathrm{imp}}$≈ 20 dB, MSE < 0.000005 (for 5 dB I/P SNR)
DNN based on the improved DAE and wavelet transform (WT) [70]	MIT-BIH	103, 105, 111, 116, 122, 205, 213, 219, 223 and 230	SNR = 23.89 dB, RMSE = 0.025 (for 5 dB I/P SNR and record 103)
Fractional Stockwell transform (FrST) [79]	MIT-BIT	100 and 222	${\mathrm{SNR}}_{\mathrm{imp}}$ = 25.0793 dB, RMSE = 0.0397 (for record 100)

**Table 2:** Summary of BW denoising on ECG signal (B).

Methods	Database	Record	Evaluation Parameters
Deep neural network [80]	MIT-BIT	103, 105, 111, 116, 122, 205, 213, 219, 223 and 230	SNR = 20.636 dB, RMSE = 0.0446 (for 1.25 dB I/P SNR and avg.)
CNN and discrete wavelet transform [81]	MIT-BIH	103, 105, 111, 116, 122, 205, 213, 219, 223, 230	SNR = 7.713 dB, RMSE = 0.294 (for 0 dB I/P SNR and avg.)
CNN and stationary wavelet transform and transformer encoder [82]	MIT-BIH	103, 105, 111, 116, 122, 205, 213, 219, 223, 230	SNR = 29.07 dB, RMSE = 0.017 (for 0 dB I/P SNR)
U-Net [73]	PTB-XL, CPSC2018	Manually label	SNR = 19.51 dB, RMSE = 0.0132 (for 0 dB I/P SNR)
Mamba [83]	QT database	-	MSE = 0.3445, PRD = 36.861%
Bidirectional gated recurrent units [74]	Experimental data collected using wearable sensors	-	${\mathrm{SNR}}_{\mathrm{imp}}$ = 20.6 dB, RMSE = 0.024, PRD = 5.5%

**Table 3:** Summary of EMG/MA denoising on ECG signal.

Methods	Database	Record	Evaluation Parameters
Successive local filtering algorithm [97]	MIT-BIH	101, 103, 113, 115, 203, 207, 208, 213	${\mathrm{SNR}}_{\mathrm{imp}}$ > 8 dB
Variable-frequency complex demodulation algorithm [59]	MIT-BIH and NSTDB	100, 101, 103, 105, 106, 115, 215, 230, Self-acquired data	${\mathrm{SNR}}_{\mathrm{imp}}$ = 4.17 dB, PRD = 5.79%
Stationary wavelet total variation algorithm [102]	MIT-BIH	100, 103, 105, 113, 115, 117, 119, 122, 200, 215, 213, 230, 231, 234	SNR = 25.44 dB, RMSE = 0.3940, PRD = 40%
Periodic non-local means filter [92]	MIT-BIH	100, 103, 104, 105, 106, 115, 215	${\mathrm{SNR}}_{\mathrm{imp}}$ = 5.804 dB, MSE = 0.0020, PRD > 15% (for 10 dB I/P SNR)
Fractional filtering and zero-phase filtering and parallel-type filter [103]	MIT-BIH	115	SNR = 13.6817 dB, MSE = 0.0146
Adaptive dual-threshold filter and discrete wavelet transform [104]	MIT-BIH	115	MSE = 0.0069
Discrete wavelet transform and electrophysiological morphology [90]	MIT-BIH	All 48 records	${\mathrm{SNR}}_{\mathrm{imp}}$ = 7.55 dB (for 10 dB I/P SNR)
Riemann–Liouville fractional integral filtering and Savitzky–Golay filtering and EMD [76]	MIT-BIH	115	SNR = 10.6116 dB, MSE = 0.0013
EMD and adaptive switching mean filter empirical [98]	MIT-BIH	100, 101, 103, 105, 115, 200, 215, 230	${\mathrm{SNR}}_{\mathrm{imp}}$ = 8.7879 dB, MSE = 0.00232, PRD = 11.5257% (for 10 dB I/P SNR)
Real-time accurate thresholding method and discrete wavelet transform [58]	MIT-BIH	233	${\mathrm{SNR}}_{\mathrm{imp}}$ = 1.16789 dB, RMSE = 0.12233
CNN and discrete wavelet transform [81]	MIT-BIH	103, 105, 111, 116, 122, 205, 213, 219, 223, 230	SNR = 7.738 dB, RMSE = 0.292 (for 0 dB I/P SNR and avg.)
CNN and stationary wavelet transform and transformer encoder [82]	MIT-BIH	103, 105, 111, 116, 122, 205, 213, 219, 223, 230	SNR = 28.11 dB, RMSE = 0.021 (for 0 dB I/P SNR)
U-Net [73]	PTB-XL, CPSC2018	Manually label	SNR = 16.76 dB, RMSE = 0.0182 (for 0 dB I/P SNR)
Bidirectional gated recurrent units [74]	Experimental data collected using wearable sensors	-	${\mathrm{SNR}}_{\mathrm{imp}}$ = 19.2 dB, RMSE = 0.029, PRD = 6.4%

**Table 4:** Summary of PLI denoising on ECG signal.

Methods	Database	Record	Evaluation Parameters
Variable-frequency complex demodulation (VFCDM) algorithm [59]	MIT-BIH and wearable armband ECG data and NSTDB	100, 101, 103, 105, 106, 115, 215, 230	${\mathrm{SNR}}_{\mathrm{imp}}$ = 4.17 dB, PRD = 5.79%, WEDD = 4.82% (for 20 dB I/P SNR)
Singular spectrum analysis [134]	Simulated data	-	Eave = 0.004
Singular spectrum analysis and digital filtering [22]	MIT-BIH	115	Eave = 31.9, MSE = 0.000007
Stationary wavelet transform (SWT) [6]	MIT-BIH	100, 101, 102, 103, 104, 105, 109, 112, 117, 118, 123, 200, 205, 213, 221, 231, and 234	${\mathrm{SNR}}_{\mathrm{imp}}$ = 49.35 dB, RMSE = 0.0006, PRD = 0.254 (for record 100 at 14.32 dB I/P SNR)
Fourier decomposition method (FDM) [40]	MIT-BIH	100, 101, 103, 105, 109, 111, 112, 113, 115, 116, 117, 118, 122, 123, 210, and 212	${\mathrm{SNR}}_{\mathrm{out}}$ = 28.1 dB, PRD > 0% (for 5 dB I/P SNR and record 100)
Fractional filtering and zero-phase filtering and parallel-type filter [103]	MIT-BIH	115	SNR = 14.2565 dB, MSE = 0.0128
Adaptive dual-threshold filter and discrete wavelet transform [104]	MIT-BIH	115	MSE = 0.0015
Biorthogonal wavelet transform and adaptive slope prediction-based threshold [72]	BMDMC	16 records	SNR = 30.0051 dB, MSE = 0.0008
Riemann–Liouville fractional integral filtering and Savitzky–Golay (SG) filtering and EMD [76]	PTB	80 + 365 records	SNR = 12.0526 dB, MSE = 0.0096
EMD and adaptive switching mean filter (ASMF) [98]	MIT-BIH	100, 101, 103, 105, 115, 200, 215, and 230	${\mathrm{SNR}}_{\mathrm{imp}}$ > 10 dB, MSE ≈ 0.002, PRD < 10% (for 10 dB I/P SNR)
Eigenvalue decomposition of the Hankel matrix [77]	MIT-BIH	100, 101, 102, 103, 104, 105, 109, 112, 117, 118, 123, 200, 205, 213, 221, 231, and 234	SNR = 49.35 dB, RMSE = 0.0006, PRD = 0.254 (for record 100 at 14.32 dB I/P SNR)
Particle swarm optimization and wavelet transform [117]	MIT-BIH	100, 102, 103, 105, 109	SNR = 19.74 dB, MSE = 0.0014, RMSE = 0.0373, PRD = 10.33% (for record 100 at 5 dB I/P SNR)
Variational mode decomposition and notch filter [129]	MIT-BIH	100, 101, 103, 105, 108, 109, 111, 112, 113, 115, 116, 117, 118, 121, 122, 123, 210, and 212	${\mathrm{SNR}}_{\mathrm{out}}$ = 21.09 dB, CC = 0.9898 (for 5 dB I/P SNR and record 100)

## Abbreviations

AWGN	Additive White Gaussian Noise: A type of noise with a constant intensity across all frequencies.
CC	Correlation Coefficient: A measure of how strongly two variables are related.
CNN	Convolutional Neural Network: A type of deep learning model often used for image and signal processing.
CVD	Cardiovascular Disease: Diseases that affect the heart and blood vessels.
DAEs	Denoising Autoencoders: Neural networks used to remove noise from signals or images.
ECG	Electrocardiography: A test that measures the electrical activity of the heart.
EKF	Extended Kalman Filter: A method used to estimate the state of a system when there is noise.
EMs	Electrode Motion Artifacts: Noise caused by electrode movement during measurements.
EMD	Empirical Mode Decomposition: A technique used to break down signals into simpler components.
EMG	Electromyogram: A test that measures the electrical activity in muscles.
GAN	Generative Adversarial Network: A model where two neural networks work together to generate realistic data.
ICA	Independent Component Analysis: A method used to separate mixed signals into independent sources.
IMF	Intrinsic Mode Function: Basic components of a signal derived through EMD.
MFMs	Magnetic Field Maps: Visual representations of magnetic fields.
MCG	Magnetocardiography: A test that records the magnetic fields generated by the heart.
MAs	Muscle Artifacts: Noise from muscle activity that interferes with signal measurements.
MSE	Mean Squared Error: A metric that measures the average squared difference between actual and predicted values.
NLM	Non-Local Means: A method for reducing noise by averaging similar data points.
OPM	Optically Pumped Magnetometer: A device used to measure small magnetic fields.
PCD	Pseudocurrent Density: A representation of current flow patterns based on magnetic data.
PCA	Principal Component Analysis: A technique used to reduce the dimensions of data by finding important patterns.
PLI	Power Line Interference: Noise from electrical power systems in signal recordings.
PRD	Percentage Root-Mean-Square Difference: A measure of the difference between two signals.
RSM	Residual Signal Method: A way to isolate and analyze noise in signals.
SNR	Signal-to-Noise Ratio: A measure of signal strength compared to noise.
SQUID	Superconducting Quantum Interference Device: A highly sensitive tool for measuring magnetic fields.
SSA	Singular Spectrum Analysis: A method for breaking down time series data into simpler parts.
SSS	Signal Space Separation: A method for separating signal from noise in magnetoencephalography and magnetocardiography.
SWT	Stationary Wavelet Transform: A method for analyzing signals without losing time information.
VMD	Variational Mode Decomposition: A method for separating signals into different modes for analysis.
